# An IgM-like inhalable ACE2 fusion protein broadly neutralizes SARS-CoV-2 variants

**DOI:** 10.1038/s41467-023-40933-3

**Published:** 2023-08-25

**Authors:** Juan Liu, Fengfeng Mao, Jianhe Chen, Shuaiyao Lu, Yonghe Qi, Yinyan Sun, Linqiang Fang, Man Lung Yeung, Chunmei Liu, Guimei Yu, Guangyu Li, Ximing Liu, Yuansheng Yao, Panpan Huang, Dongxia Hao, Zibing Liu, Yu Ding, Haimo Liu, Fang Yang, Pan Chen, Rigai Sa, Yao Sheng, Xinxin Tian, Ran Peng, Xue Li, Junmian Luo, Yurui Cheng, Yule Zheng, Yongqing Lin, Rui Song, Ronghua Jin, Baoying Huang, Hyeryun Choe, Michael Farzan, Kwok-Yung Yuen, Wenjie Tan, Xiaozhong Peng, Jianhua Sui, Wenhui Li

**Affiliations:** 1 National Institute of Biological Sciences, Beijing, China; 2 Huahui Health Ltd, Beijing, China; 3 National Kunming High-level Biosafety Primate Research Center, Institute of Medical Biology, Chinese Academy of Medical Sciences and Peking Union Medical College, Yunnan, China; 4 Department of Microbiology, School of Clinical Medicine, Li Ka Shing Faculty of Medicine, The University of Hong Kong, Hong Kong Special Administrative Region, China; 5 State Key Laboratory of Emerging Infectious Diseases, Li Ka Shing Faculty of Medicine, The University of Hong Kong, Hong Kong Special Administrative Region, China; 6 Department of Clinical Microbiology and Infection Control, The University of Hong Kong-Shenzhen Hospital, Shenzhen, Guangdong Province China; 7 Carol Yu Centre for Infection, Li Ka Shing Faculty of Medicine, The University of Hong Kong, Hong Kong Special Administrative Region, China; 8 Centre for Virology, Vaccinology and Therapeutics, Hong Kong Science and Technology Park, Hong Kong Special Administrative Region, China; 9 Beijing Ditan Hospital, Capital Medical University, Beijing, China; 10 National Institute for Viral Disease Control and Prevention, Chinese Center for Disease Control and Prevention (China CDC), Beijing, China; 11 Department of Immunology and Microbiology, Scripps Research, Jupiter, FL USA; 12 State Key Laboratory of Medical Molecular Biology, Department of Molecular Biology and Biochemistry, Institute of Basic Medical Sciences, Medical Primate Research Center, Neuroscience Center, Chinese Academy of Medical Sciences, School of Basic Medicine, Peking Union Medical College, Beijing, China; 13 Tsinghua Institute of Multidisciplinary Biomedical Research, Tsinghua University, Beijing, China

**Keywords:** SARS-CoV-2, Protein delivery, Antiviral agents, Hamster

## Abstract

Many of the currently available COVID-19 vaccines and therapeutics are not effective against newly emerged SARS-CoV-2 variants. Here, we developed the metallo-enzyme domain of angiotensin converting enzyme 2 (ACE2)—the cellular receptor of SARS-CoV-2—into an IgM-like inhalable molecule (HH-120). HH-120 binds to the SARS-CoV-2 Spike (S) protein with high avidity and confers potent and broad-spectrum neutralization activity against all known SARS-CoV-2 variants of concern. HH-120 was developed as an inhaled formulation that achieves appropriate aerodynamic properties for rodent and monkey respiratory system delivery, and we found that early administration of HH-120 by aerosol inhalation significantly reduced viral loads and lung pathology scores in male golden Syrian hamsters infected by the SARS-CoV-2 ancestral strain (GDPCC-nCoV27) and the Delta variant. Our study presents a meaningful advancement in the inhalation delivery of large biologics like HH-120 (molecular weight (MW) ~ 1000 kDa) and demonstrates that HH-120 can serve as an efficacious, safe, and convenient agent against SARS-CoV-2 variants. Finally, given the known role of ACE2 in viral reception, it is conceivable that HH-120 has the potential to be efficacious against additional emergent coronaviruses.

## Abstract (web summary)

In this study, the authors report the development of an inhalable IgM-like ACE2 and show that it broadly neutralizes SARS-CoV-2 variants, lowers viral loads and lung pathology in hamsters when administered early, and has good safety profiles in rats and monkeys.

## Introduction

The vast scale of coronavirus disease 2019 (COVID-19) caused by severe acute respiratory syndrome coronavirus-2 (SARS-CoV-2) and the suboptimal protection efficacy for the viral infection after vaccination have provided a fertile ground for the emergence of variants with adaptive advantages, including increased transmissibility and the ability to escape from neutralizing antibodies targeting SARS-CoV-2’s receptor-binding domain (RBD)^1^. The currently prevailing Omicron variants harbor more than thirty mutations in the S protein^2,3^, and are markedly resistant to neutralization by serum from convalescent patients or vaccinated individuals^2–4^. These variants also escape the majority of SARS-CoV-2 neutralizing antibodies of diverse epitopes^2–4^. Notably, three antibody regimens (Bamlanivimab plus Etesevimab, Casirivimab plus Imdevimab, and Sotrovimab) granted Emergency Use Authorization (EUA) by the FDA for treatment of mild-to-moderate COVID-19 in 2021 have now been limited in use for the treatment of infections caused by susceptible SARS-CoV-2 variants (i.e., not for the Omicron variant)^5^. Two small molecule oral antivirals (Molnupiravir and Paxlovid) granted EUA by the FDA in Dec. 2021 showed clinical benefit in Omicron variant-infected and hospitalized patients not requiring oxygen therapy on admission^6^. However, each has its own limitations: Molnupiravir has potential host mutagenic risk and can potentially increase SARS-CoV-2 mutation frequencies^7,8^. Paxlovid (Ritonavir-Boosted Nirmatrelvir) is contraindicated with drugs for drug-drug interactions owing to Ritonavir’s CYP3A induction effect, limiting its widespread use^9^. Moreover, a recent study reported that the effect of Paxlovid in patients under 65 is limited^10^.

Along with the mutation of the virus and the building of the population immunity, the major symptoms of COVID-19 are also evolving, from the severe acute respiratory syndrome caused by the ancestral strains through Delta variant to the more recent mild-to-moderate upper respiratory symptoms caused by the Omicron variant^11,12^. Nonetheless, the respiratory system remains the major target for the virus. Although SARS-CoV-2 can also infect other organs, this typically only occurs in relatively severe cases or in late-stage COVID-19^13^. Thus, anti-COVID-19 agents that confer their benefit directly in the upper respiratory tract and lungs, ideally through inhalation, could enable superior clinical efficacy over the systemic delivery route (e.g., intravenous administration) generally used for delivery of monoclonal antibodies or other large biologics, which are transported rather inefficiently to the lungs from circulation (concentration in the lungs is 500–10,000 times lower than that in circulation^14^).

Angiotensin converting enzyme 2 (ACE2) is the cellular receptor required for viral entry of SARS-CoV-2^15,16^ and SARS-CoV^17,18^, as well as all of their known variants and closely related viruses from animals^19^. ACE2 is a type I transmembrane protein with two extracellular domains, the amino-terminal metallo-enzyme domain and the carboxy-terminal collectrin-like domain (CLD)^20,21^. The SARS-CoV-2 S protein binds on top of the metallo-enzyme domain (independent of its enzymatic activity)^21^, after which the virus either undergoes endocytosis (as with Omicron infection)^22^ or proceeds to direct membrane fusion (with the aid of TMSSP22) in other SARS-CoV-2 infections^23^. Therefore, unlike neutralizing antibodies, soluble proteins derived from the extracellular domain of human ACE2 (hACE2) can serve as a decoy for preventing the viral binding to the host cell and, notably, are intrinsically resistant to viral mutational escape^24^.

Recombinant hACE2 proteins—with or without a tag—have been tested for blocking SARS-CoV-2 infection in cell cultures^25–27^ and in clinical trials for treatment of COVID-19. Recombinant hACE2-Fc (hACE2 fused with human IgG1 Fc domain) or other forms of engineered hACE2 fusion proteins with improved affinity can neutralize SARS-CoV-2^24,26–32^, but their antiviral potency in human trials remains to be studied. Since SARS-CoV-2 viral entry is mediated by an efficient coordinated binding of the trimeric S protein to ACE2 expressed on the host cell surface, the recombinant hACE2 variants designed to act as a decoy of SARS-CoV-2 needs to possess high binding activity to block viral binding to the receptor.

Here, we developed a soluble hACE2 molecule into an IgM-like multivalent Fc-fusion protein: HH-120. Single-particle negative-stain electron microscopy (EM) revealed that HH-120 molecules mainly exist as pentamers and hexamers. HH-120 binds to the S protein of SARS-CoV-2 with high avidity (>1 × 10^−12^ M) and confers ~88–265 fold higher neutralization activity than a human IgG1 Fc-tagged bi-valent ACE2 (hACE2-hIgG1) against ancestral strain (IVDC-QD-11-2P2) infection of Vero cells, with an IC_50_ (half maximal inhibitory concentrations) of 20–60 ng/mL (IC_90_ of 180–540 ng/mL). HH-120 confers broad-spectrum and potent neutralization activity against all tested (more than 30) pseudotyped SARS-CoV-2 variants, including the Omicron variants. Importantly, HH-120 can be formulated as an aerosol inhalation for direct pulmonary delivery, thus allowing rapid action, high local concentration in respiratory tract (especially in lungs), and minimal adverse systemic effects. In golden Syrian hamster infection models with the ancestral strain (GDPCC-nCoV27) and Delta variant SARS-CoV-2 virus, inhalation of aerosolized HH-120 for early treatment resulted in potent antiviral effects, reducing lung pathology scores and leading to ~3 log reductions in viral loads (assessed as genomic RNA (gRNA), subgenomic RNA (sgRNA), and infectious viral titers). Preclinical toxicological studies in rats and cynomolgus monkeys demonstrate that HH-120 can be safely inhaled, supporting further clinical development of HH-120 into a safe, convenient, and efficacious agent fighting against variants of SARS-CoV-2 that cause COVID-19, and potential threats caused by additional ACE2-utilizing coronaviruses that may emerge in the future.

## Results

### HH-120, an IgM-like ACE2-Fc fusion protein assembled as pentamers and hexamers strongly binds to the SARS-CoV-2 receptor-binding domain (RBD)

**Fig. 1:**
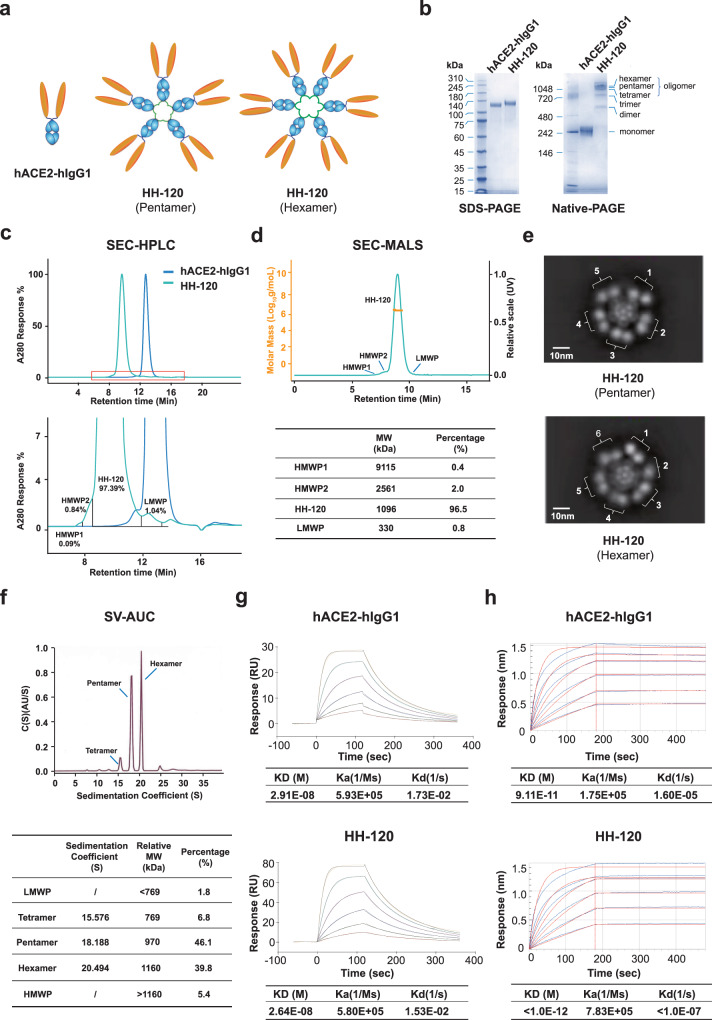
Engineering and physicochemical characterization of HH-120. **a** Schematic diagram showing a hACE2-hIgG1 molecule and two forms of HH-120 molecules in pentamer and hexamer forms. Orange: extracellular domain of hACE2, Blue: hIgG1 Fc, Green: IgM tailpiece. **b** SDS-PAGE and Native-PAGE analyses of purified HH-120 and hACE2-hIgG1 protein samples. **c** SEC-HPLC analysis of HH-120 and hACE2-hIgG1. 100 μg of HH-120 or hACE2-hIgG1 samples were run on a TSKgel G4000SWxl analytical column using an Agilent 1260 HPLC system. The lower panel presents the magnification of the red box zone in the upper HPLC chromatogram. HMWP1, HMWP2, HH-120, LMWP components, and their percentages are indicated. **d** SEC-MALS chromatograms with calculated MWs. The cyan chromatogram shows the normalized UV (280 nm) signal, and the orange line is the MW of material eluted at the time indicated. The table shows the MW and the percentage of each component determined by SEC-MALS. **e** Single-particle negative-stain EM analysis of HH-120. Representative EM images of a HH-120 pentamer molecule and a hexamer molecule are shown. **f** HH-120 characterization using SV-AUC. The sedimentation coefficient, relative MW, and the percentage of each component determined by SV-AUC are shown. **g** Affinity and kinetic characterization of the binding of HH-120 or hACE2-hIgG1 to SARS-CoV-2 RBD using a Biacore T200 instrument. **h** Binding avidity of HH-120 or hACE2-hIgG1 to SARS-CoV-2 RBD using a Fortebio RED384 instrument. All data shown are representatives of at least three independent experiments (**b**–**h**). Source data are provided as a Source Data file.

Seeking to identify a candidate molecule with strong virus neutralization activity and good druggability, we constructed and screened a panel of Fc-tagged-hACE2-derived molecules with diverse valency (from monomer to twelve-unit oligomers). The screening comprised RBD binding affinity assays, pseudovirus neutralization assays, and analyses of physicochemical properties including expression level, homogeneity, stability, and feasibility of purification. Finally, an IgM-like hACE2-Fc fusion protein named HH-120 was selected for its combined features of potent neutralization activity and desired physicochemical properties. In the neutralization assays, HH-120 confers stronger activity than other forms of ACE2 fusion proteins including hACE2-hIgG1, a hACE2 tandem fusion protein—hACE2-hACE2-hIgG1, a fusion protein with hACE2 fused to the C terminus and N terminus of hIgG1—hACE2-hIgG1-hACE2, fusion proteins with hACE2 fused to the C terminus of monoclonal antibodies targeting S protein (Supplementary Fig. 1). HH-120 contains the extracellular domain of human ACE2 (amino acid 19–615). Since the Zinc metallo-enzyme activity of ACE2 is not involved in viral infection^18,21^, H374N and H378N mutations of ACE2 were introduced to HH-120 to deactivate any Zinc metallo-enzyme activity^17^, thus precluding possible safety risks with enzyme-activity-related physiological disorders. The Fc portion of HH-120 is derived from the human IgG1 Fc fragment, fused with an IgM tailpiece (PTLYNVSLVMSDTAGTCY) that enables the pentamerization or hexamerization of the fusion protein^33^, thus resulting in an IgM-like molecule (Fig. 1a). The cysteine in the IgM tailpiece allows the formation of inter-monomeric disulfide bridges that facilitate polymerization, as has been demonstrated for other previously reported IgM-like molecules^34^. ACE2 is connected to Fc through a flexible linker, and only one pair of inter-monomer disulfide bonds is formed between monomers.

HH-120 molecules were produced in Chinese hamster ovary (CHO) cells, and after multiple steps of purification, the final yield reached around 0.5–0.6 g/L and the purity reached >96% as determined by size exclusion high performance liquid chromatography (SEC-HPLC) analysis and size-exclusion chromatography coupled with multi-angle light scattering (SEC-MALS). The purified HH-120 showed as one major band with an apparent MW of around 150 kDa on reducing SDS-PAGE (Fig. 1b). Native-PAGE revealed three adjacent bands with an apparent MW of around 1000 kDa, likely representing hexamers, pentamers, and tetramers, respectively, collectively referred to as “Oligomer”; Native-PAGE also revealed two bands with sizes corresponding to theoretical MW of trimers and dimers, probably formed owing to the heating procedure caused oligomeric disassembly (Fig. 1b). The purity of HH-120 oligomer ranged from 96.2% to 99.5% with different batches when analyzed using SEC-HPLC. One batch showed a 97.39% purity represented by a main peak in SEC-HPLC; the impurities were <3% and included two small peaks for high molecular weight proteins (HMWPs) and a small peak for low molecular weight proteins (LMWPs) (Fig. 1c).

SEC-MALS demonstrated HH-120 elutes as one main peak (96.5%) exhibiting an average MW of 1096 kDa, within the range of the theoretical MW of pentamer (969 kDa) and hexamer (1163 kDa). Consistent with the SEC-HPLC data, SEC-MALS also showed a small amount of two types of aggregates: one with a MW of 2561 kDa accounting for 2.0% and the other one with MW of 9115 kDa accounting for 0.4%, respectively (Fig. 1d). In addition, SEC-MALS indicated a LMWP (0.8%) impurity with a MW of 330 kDa, a value between the theoretical MW of the monomer (193.8 kDa) and the dimer (387.6 kDa).

We next prepared negatively stained single-particle samples of purified HH-120 and examined the compositional integrity and conformational states of HH-120 by EM. We observed that HH-120 molecules mainly exist in the form of pentamer and hexamer in solution, appearing as 5 or 6 of petals in a flower-like annular core (formed by Fc Tags), and 10 or 12 surrounding spheres (formed by each ACE2 domain), each Fc petal associates with two ACE2 spheres (Fig. 1e). There is also a small amount of tetramer, probably formed owing to incomplete disulfide bond formation. Using sedimentation velocity—analytical ultracentrifugation (SV-AUC), we further quantified the proportions of hexamer, pentamer, and tetramer in purified HH-120 were 39.8%, 46.1%, and 6.8%, respectively (Fig. 1f).

The binding affinity and avidity of HH-120 were measured and compared with bivalent hACE2-hIgG1 using Surface Plasmon Resonance (SPR, BIAcore T200) and Bio-Layer Interferometry (BLI, Fortebio Octet RED384) technologies, respectively. The monovalent binding affinity of HH-120 to SARS-CoV-2 RBD (amino acids 316–512 of the S protein in the SARS-CoV-2 D614 strain) is about 2.64 × 10^−8 ^M, which is comparable to that of hACE2-hIgG1 (2.91 × 10^−8 ^M) binding to the RBD (Fig. 1g). Consistent with the IgM-like format design of HH-120, the multivalent binding avidity of HH-120 is higher than 1.0 × 10^−12 ^M (exceeding the detection limit of the instrument) and is at least 90-fold higher than hACE2-hIgG1 (9.1 × 10^−11 ^M) in binding to the RBD (Fig. 1h). The binding affinities of HH-120 to S trimer protein of the Alpha, Beta, Delta, and Omicron variants were also measured by SPR: HH-120 binds to these four variants with similar affinity, having KD values around 1 nM for each (Supplementary Fig. 2).

### Enhanced potency of HH-120 over hACE2-hIgG1 for inhibiting syncytia formation

Severe cases of COVID-19 are associated with extensive lung damage, including the presence of infected multinucleated syncytial pneumocytes^35^ caused by membrane fusion of SARS-CoV-2 infected cells expressing S proteins at the cell surface and ACE2-positive neighboring cells. The Delta variant causes increased disease severity compared with earlier strains, which is thought to be at least partially associated with increased syncytium formation^36^. We used in vitro cell-cell membrane fusion assays to test whether HH-120 can inhibit SARS-CoV-2 induced membrane fusion.

**Fig. 2:**
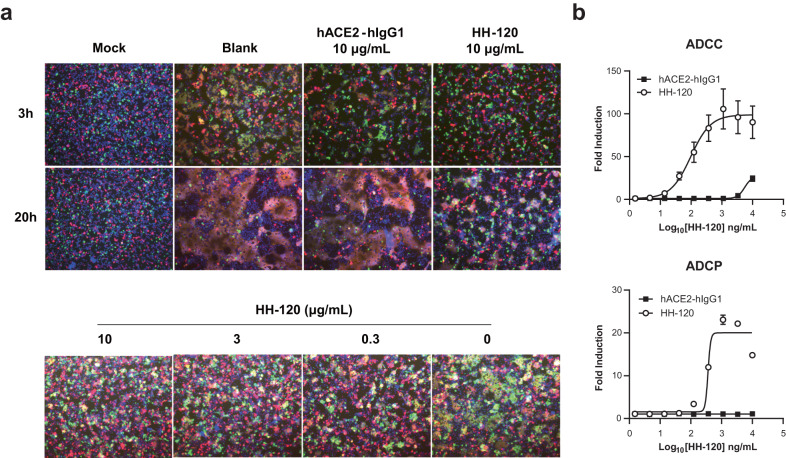
hACE2-hIgG1 and HH-120 comparison in syncytia formation inhibition, ADCC, and ADCP activities. **a** HH-120 inhibited syncytia formation. Syncytia formation assay was performed by co-culturing 293T cells co-expressing the SARS-CoV-2 S protein and EGFP (S cells) with 293T cells co-expressing hACE2 and mCherry (ACE2 cells) at a 1:1 ratio. S-ACE2 interaction-mediated syncytia formation, as reflected by polykaryocytes and the mergers of green and red cells, was observed under a fluorescent microscope (20×). Mock, co-culturing 293T cells transfected with the EGFP plasmid alone with ACE2 cells; Blank, co-culturing of S cells and ACE2 cells with medium only. Green, S cells; red, ACE2 cells; blue, cell nucleus. **b** HH−120 mediates ADCC and ADCP against CHO-S cells. Target cells (T) (CHO-S cells) were pre-incubated with serially diluted HH-120 or hACE2-hIgG1, effector cells (E) (Jurkat-NFAT-luc2p/FcγRIIIa (F158) cells for ADCC or Jurkat-NFAT-luc2p/FcγRIIa (R131) cells for ADCP) were added at an E:T ratio of 6:1. The ADCC and ADCP activities are shown as fold induction of luciferase activity over blank control. Data shown are average values of three replicates from distinct samples. Error bars represent the SD. Data shown are representatives of three independent experiments (**a**, **b**). Source data are provided as a Source Data file.

293T cells expressing SARS-CoV-2 S protein fused with EGFP and hACE2 tagged with mCherry were co-incubated for 3 h or 20 h with the various concentrations of HH-120 or hACE2-hIgG1 protein. Cell membrane fusion mediated by S protein and ACE2 manifest as formation of polykaryocytes (multinucleated syncytia) recorded using fluorescent microscopy. Syncytia formation was evident after the 3 h co-incubation, and most cells had fused to each other by 20 h. In the presence of 10 μg/mL HH-120, the number of syncytia was reduced by more than 90%; in contrast, hACE2-hIgG1 only weakly inhibited syncytia formation under the same condition (Fig. 2a upper panel), consistent with HH-120’s high binding avidity over hACE2-hIgG1. HH-120 inhibited syncytium formation in a dose-dependent manner, and a low concentration of 0.3 μg/mL of HH-120 still conferred potent inhibition activity (Fig. 2a bottom panel). These results show that HH-120 can effectively block syncytia formation induced by SARS-CoV-2.

### HH-120 has enhanced ADCC and ADCP activities over hACE2-hIgG1

Neutralizing antibodies with intact Fc-enabled ADCC (antibody-dependent cellular cytotoxicity) and ADCP (antibody-dependent cellular phagocytosis) activity have been shown to be more effective than their corresponding Fc null format versions in terms of antiviral activity in a mouse model of SARS-CoV-2 infection^37^. For HH-120, although it contains intact Fc fragments, it was not clear if the IgM-like format affects its Fc function. We therefore employed bioluminescent-based reporter bioassays to evaluate the ADCC and ADCP activities mediated by HH-120. Using FcγR-expressing Jurkat/NFAT-luc2p/FcγRIIIa (F158) or Jurkat/NFAT-luc2p/FcγRIIa (R131) cells as the effector cells and using CHO cells expressing SARS-CoV-2 S protein (CHO-S) as the target cells in these reporter bioassays, we found that HH-120 treatment induced dose-dependent ADCC and ADCP activities against CHO-S cells. Compared to hACE2-hIgG1, HH-120 induced ~100-fold stronger ADCC and at least ~20-fold stronger ADCP activities (Fig. 2b). These results indicate that the IgM-like design of HH-120 has no negative effect on its Fc function, and support that HH-120’s multivalency enhances both ADCC and ADCP activities.

### HH-120 has broad-spectrum neutralization activity against SARS-CoV-2 viruses

**Fig. 3:**
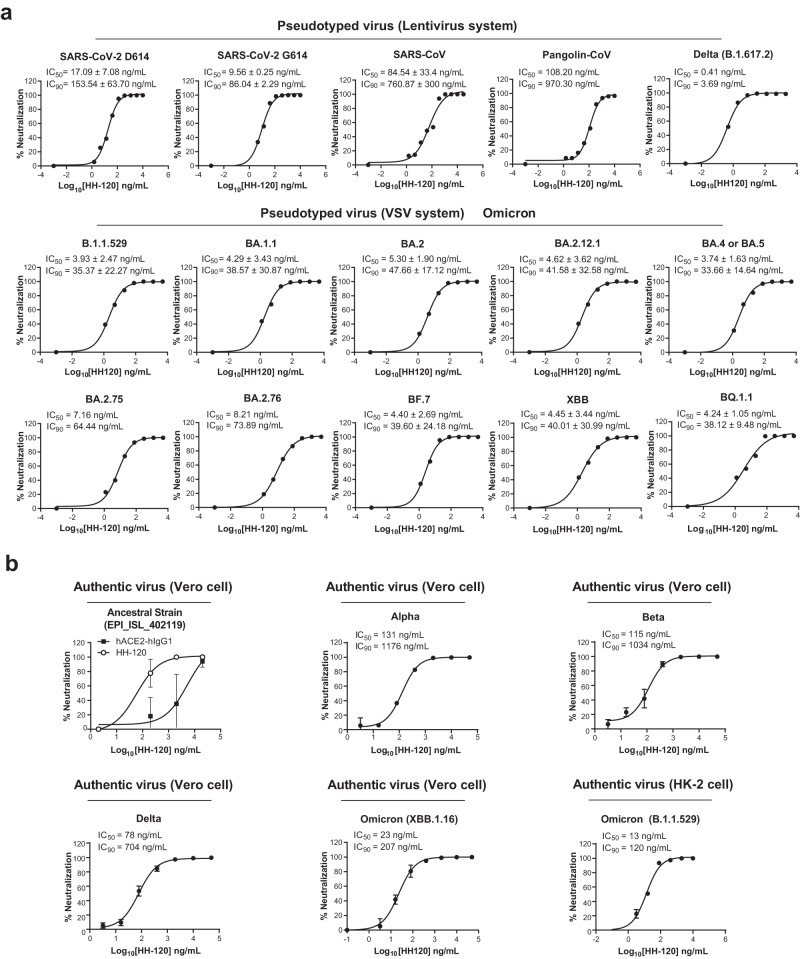
Neutralization activity of HH-120 against SARS-CoV-2 and other coronaviruses. **a** Neutralization of pseudotyped viruses (lentivirus or VSV system) of the SARS-CoV-2 D614 strain, the G614 strain, SARS-CoV, Pangolin-CoV, Delta (B.1.617.2) variant, Omicron (B.1.1.529, BA.1.1, BA.2, BA.2.12.1, BA.4 or BA.5, BA.2.75, BA.2.76, BF.7, XBB, BQ.1.1) variants by HH-120. Pseudotyped viruses were incubated with serially diluted HH-120 (1.5–10,000.0 ng/mL), then inoculated with 293T-hACE2 cells. Results shown are representative data of at least two independent experiments, except for the Pangolin-CoV pseudotyped virus. Data shown are average values of two replicates from distinct samples, except for the Pangolin-CoV pseudotyped virus without replicates. **b** Neutralization of SARS-CoV-2 authentic virus of ancestral strain (EPI_ISL_402119), Alpha, Beta, Delta, Omicron (XBB.1.16 and B.1.1.529 sublineages) variants by HH-120. Vero cells or HK-2 cells were used as host cells. Results shown are representative data of at least two independent experiments. Data shown are average values of three replicates from distinct samples. Error bars represent the SD. Cells without virus infection were used as assay blanks; calculated IC_50_ and IC_90_ values were determined using 4-parameter logistic regression (**a**, **b**). Source data are provided as a Source Data file.

To evaluate the broadness of HH-120’s capacity to neutralize SARS-CoV-2 variants, pseudotyped SARS-CoV-2 variants (lentivirus-based or vesicular stomatitis virus (VSV) -based, enveloped with S protein from different variants) were generated and used for testing HH-120’s neutralization activities. HH-120 showed a dose-dependent neutralization activities against the SARS-CoV-2 D614 strain, the SARS-CoV-2 G614 strain, SARS-CoV, Pangolin-CoV, Delta (B.1.617.2) variant pseudotyped viruses (lentivirus-based) with IC_50_ values ranging from 0.41–108.20 ng/mL and IC_90_ values ranging from 3.69–970.30 ng/mL, and Omicron (B.1.1.529, BA.1.1, BA.2, BA.2.12.1, BA.4 or BA.5, BA.2.75, BA.2.76, BF.7, XBB, BQ.1.1) variant pseudotyped viruses (VSV-based) with IC_50_ values ranging from 3.74 to 8.21 ng/mL and IC_90_ values ranging from 33.66 to 73.89 ng/mL (Fig. 3a). Moreover, a series mutant pseudotyped viruses with mutation-containing S proteins were produced, including 16 G614 variants (each containing a single mutation in the S protein), as well as other variants on the WHO list. HH-120 exerted potent inhibition activities for all of these pseudotyped viruses, with IC_50_ values ranging from 0.24 to 5.30 ng/mL and IC_90_ values ranging from 2.16 to 47.70 ng/mL (Supplementary Table 1).

Subsequently, we conducted neutralization experiments using authentic SARS-CoV-2 viruses. Specifically, 200 TCID_50_ (Median Tissue Culture Infectious Dose) of SARS-CoV-2 authentic live viruses (an ancestral strain and multiple variants) were mixed with serially diluted HH-120 or hACE2-hIgG1, followed by addition to Vero cell cultures (or otherwise specifically stated) for infection and cytopathic effect (CPE) observation^38^. Quantification of viral RNA in infected cells was analyzed by qRT-PCR. HH-120 had an IC_50_ of 20–60 ng/mL (IC_90_ = 180–540 ng/mL) in neutralizing against infection by a SARS-CoV-2 ancestral strain (EPI_ISL_402119), which is about 88–265-fold more potent than that of hACE2-hIgG1 (IC_50_ = 5300 ng/mL, IC_90_ = 47700 ng/mL) (Fig. 3b). Recall that the multivalent binding avidity of HH-120 is at least 90-fold higher than hACE2-hIgG1 (9.1 × 10^−11 ^M) in binding to the RBD in a BLI assay (Fig. 1f). The higher neutralization potency of HH-120 is consistent with its higher binding avidity. HH-120 also potently inhibited the infection of the SARS-CoV-2 Alpha variant, the Beta variant, and the Delta variant with respective IC_50_ values of 131 ng/mL, 115 ng/mL and 78 ng/mL, and respective IC_90_ values of 1176 ng/mL, 1034 ng/mL and 704 ng/mL (Fig. 3b). HH-120 also demonstrated neuralization activity against the Omicron variant. HH-120 inhibited the infection of the Omicron sublineage XBB.1.16 in a dose-dependent manner with an IC_50_ value of 23 ng/mL, and an IC_90_ value of 207 ng/mL (Fig. 3b). In a separate authentic virus neutralization assay using HK-2 as host cells, HH-120 also demonstrated similar neutralization activity against the Omicron sublineage B.1.1.529 with an IC_50_ value of 13 ng/mL, and an IC_90_ value of 120 ng/mL (Fig. 3b). These results demonstrate HH-120 has potent and broad-spectrum neutralization activity against infection of SARS-CoV-2 variants (including Omicron).

### HH-120 can be effectively nebulized using a mesh vibrating nebulizer

**Fig. 4:**
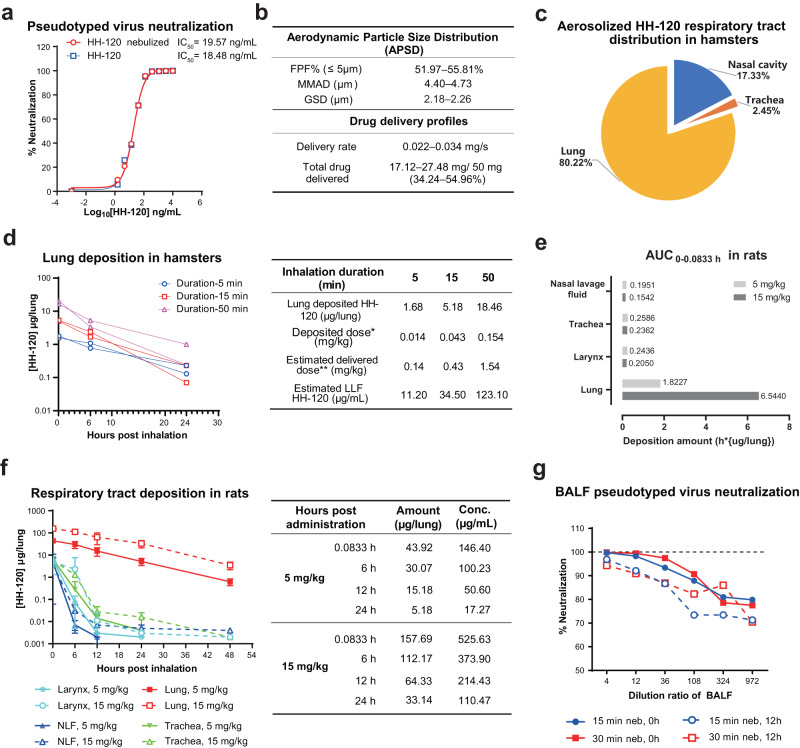
Inhalation delivery assessment of HH-120. **a** Pseudotyped virus neutralization analysis of HH-120 before and after nebulization. HH-120 aerosols were generated by nebulizing 5 mL of 10 mg/mL HH-120 solution. Data shown are average values of two replicates from distinct samples. **b** APSD profiles and drug delivery profiles (delivery rate and total drug delivered) after inhalation of HH-120 measured by an NGI and a breathing simulator. Data shown are the ranges of six independent experiments. FPF fine particle fraction, MMAD mass median aerodynamic diameter, GSD geometric standard deviation. **c** HH-120’s deposition profiling in hamster respiratory tract immediately after the inhalation (*n* = 2). **d** Clearance kinetics of inhaled HH-120 in hamster lungs after inhalation at 0, 6, and 24 h after inhalation (*n* = 2) (left panel). Each curve represents data from one hamster. Body weight-based lung deposited doses, estimated delivered doses and estimated concentration of HH-120 in lung lining fluid (LLF) for the three dose groups are shown in the right panel. * the calculation was based on the estimation of the surface area of mammalian lungs as 1 m^2^/kg body weight^45^; ** the delivered dose is about 10-fold of the deposited dose in rodents^46^, LLF volume for hamsters was estimated as 0.15 mL. **e** AUC values of 0–5 min (0.0833 h) of inhaled HH-120 in rat lungs after inhalation (*n* = 6 per group). **f** Clearance kinetics of inhaled HH-120 in rat nasal cavities, larynxes, tracheas, and lungs after inhalation (*n* = 6 except *n* = 4–6 for 48 h timepoint) (left panel), error bars represent the SD. HH-120 amounts deposited in the lungs and estimated concentration of HH-120 in LLF at 0.0833, 6, 12, and 24 h after inhalation are shown in the right panel, LLF volume for rats was estimated as 0.3 mL. **g** Neutralization activity analysis of HH-120 after inhalation (*n* = 2/group). Data shown are the mean values **(c**, **e–g**). HH-120 aerosols were generated by nebulizing 5 mg/mL HH-120 via Aerogen solo nebulizer administered to hamsters using a whole-body inhalation exposure system (**c**–**d**) Ancestral D614 strain pseudotyped viruses were used (**a**, **g**). Source data are provided as a Source Data file.

In treating COVID-19, systemic application of drugs—and especially biologics of high MW—may be suboptimal for bioavailability in the lungs^14^. Several IgG or IgM monoclonal antibodies are being developed for the prevention or treatment of COVID-19 by aerosol or nasal administration^39–41^. Seeking to maximize the exposure of HH-120 to SARS-CoV-2 viruses to improve its bioavailability, we evaluated whether HH-120 could be administrated via inhalation, which should support direct delivery of HH-120 (with a MW of ~1000 KDa) to the entire respiratory tract from the nose to lung, thus efficiently targeting the SARS-CoV-2 virus locally at the main infection sites. We choose a mesh vibrating nebulizer instrument (Aerogen Solo) that employs a mini pump to produce fine particles with geometric diameter range between 1–5 μm^42^ (i.e., ideally sized particles for deep lung deposition^43^). To evaluate the influence of nebulization on the physicochemical properties and neutralization activity of HH-120, HH-120 aerosols produced by the Aerogen Solo instrument were collected and analyzed: HH-120 exhibited the same SEC-HPLC peak profiles before and after nebulization, without notable aggregation or degradation, and the HH-120 main peak maintained at a high percentage level after nebulization (98.78%) as compared to that before nebulization (99.13%) (Supplementary Fig. 3). Moreover, importantly, HH-120 had the same binding avidity (Supplementary Fig. 4) and neutralization activity before and after nebulization (Fig. 4a).

Aerosol droplets ranging from 0.5 to 5 μm in diameter have been reported as the most suitable particle sizes for deposition in small airways and alveoli in humans^43^. We next characterized HH-120 aerodynamic particle size distribution (APSD) using Next Generation Impactor (NGI), a high-performance cascade impactor. The fine particle fraction (FPF) (≤5 μm) of HH-120 aerosols ranged from 51.97–55.81%, with a mass median aerodynamic diameter (MMAD) of 4.40–4.73 μm and a geometric standard deviation (GSD) of 2.18–2.26 μm (Fig. 4b), values within suitable ranges for lung deposition in both human and rodents^43,44^. Detailed particle size distribution in different size ranges is presented in Supplementary Table 2. We also used a breathing simulator—an instrument designed to generate and apply an inhalation and/or exhalation profile that mimics a human subject—to assess the drug delivery rate and total drug delivered. In the HH-120 inhalation test, aerosols generated by nebulizing 50 mg HH-120 solution using the Aerogen Solo were delivered at a rate of 0.022–0.034 mg/s, and a total of 17.12–27.48 mg (34.24–54.96%) HH-120 were delivered, indicating that about half of HH-120 aerosols produced by Aerogel Solo could be inhaled by a human.

### HH-120 can be effectively deposited in the lungs of hamsters through inhalation

Prior to conducting in vivo efficacy studies, we analyzed the pharmacokinetic (PK) profile of aerosolized HH-120 in the respiratory tract of hamsters. Deposition of HH-120 in the respiratory tract was analyzed by measuring HH-120 amount in the ex vivo tissue samples after inhalation. HH-120 aerosols generated by nebulizing 5 mg/mL HH-120 solution were delivered to hamsters by using a whole-body inhalation exposure system. PK analysis showed that immediately after 50 min of HH-120 inhalation, about 80.22%, 17.33%, and 2.45% of inhaled HH-120 was deposited in the lung, nasal cavity, and trachea of the animals, respectively (Fig. 4c). These results support that the majority of the aerosolized HH-120 was deposited in the lungs after inhalation.

Further testing of the lung PK profile of inhaled HH-120 in three dose groups (5-min, 15-min, and 50-min inhalation of HH-120 aerosols generated by nebulizing 5 mg/mL HH-120 solution) in hamsters showed that the deposited HH-120 in the lungs was cleared after inhalation, with a half-life of about 6 h across the three dose tested groups (Fig. 4d left panel). The lung delivered doses and deposited doses were estimated. Immediately after 5-min, 15-min, and 50-min inhalation of HH-120 aerosols, total HH-120 depositions per lung were 1.68, 5.18, and 18.46 μg, respectively. According to the estimation of the surface area of mammalian lungs as 1 m^2^/kg body weight^45^, and a body weight of about 120 g per hamster, the body weight-based lung deposited doses of HH-120 for the three different inhalation duration groups were calculated as ~0.014, 0.043, and 0.154 mg/kg, respectively. Given that the deposited dose for rodents is about 10% of the delivered dose^46^, the delivered doses thus can be estimated as ~0.14, 0.43, and 1.54 mg/kg, respectively (Fig. 4d right panel).

According to the estimation of the volume of lung lining fluid (LLF) in the literature for different species (e.g., for humans the LLF volume ranging ~15–70 mL)^47,48^, and considering species differences in lung surface area and body weight^47^, the LLF volume for hamsters can be estimated as half of that of rats (0.1–0.3 mL), in a range of ~0.05–0.15 mL. Take the high-end estimation of 0.15 mL into calculation, the HH-120’s LLF concentrations in hamsters for the delivered doses of 0.14, 0.43, and 1.54 mg/kg can be estimated as ~11.20 μg/mL, 34.50 μg/mL and 123.10 μg/mL, respectively (Fig. 4d right panel). Recall the in vitro IC_90_s for authentic viruses are 120–1176 ng/mL (Fig. 3b), thus even the lowest delivered dose (0.14 mg/kg) in hamsters can provide at least 10-fold higher local HH-120 concentration than the highest IC_90_.

The PK profile of inhaled HH-120 was further evaluated in SD rats with 29 rats (14–15/sex) in each target delivered dose. HH-120 was given via an inhalation exposure system which was connected to Aerogen Solo nebulizer systems at target delivered dose levels of 5 and 15 mg/kg/dose corresponding to actual delivered doses of 4.127 and 15.000 mg/kg/dose. Blood serum samples, larynx, trachea, and lung tissues of the rats, as well as the nasal lavage fluid (NLF) were collected separately at 0 (<5 min), 6, 12, 24, and 48 h after the completion of a single dose of HH-120 inhalation (*n* = 6 except *n* = 4–6 for the 48 h timepoint). HH-120 was undetectable in serum samples using a validated ELISA assay. Consistent with the results obtained from hamsters described above, the HH-120 deposited in the lungs after inhalation was cleared with a half-life of about 8 h and it was cleared faster in other regions of the respiratory tract (Fig. 4e, f). The C_max_ of HH-120 in the lung tissues increased in an approximately dose-proportional manner (Fig. 4f left panel). Based on the surface area for mammalian lungs (~1 m^2^/kg body weight)^45^, and about 230 g average body weight for the rats used in this study, the body weight-based lung deposited dose of HH-120 at C_max_ is ~0.19 mg/kg and 0.69 mg/kg for the target delivered doses of 5 and 15 mg/kg, respectively. T_max_ was the first sampling timepoint (5 min) for all the dose group animals.

According to the previously described LLF volume estimation for rats (~0.1–0.3 mL), and take the high-end estimation of 0.3 mL into calculation, the HH-120’s LLF concentrations in rats can be estimated as 50.60 μg/mL and 17.27 μg/mL at 12 h and 24 h post administration (5 mg/kg target delivered dose), respectively; 214.43 μg/mL and 110.47 μg/mL at 12 h and 24 h post administration (15 mg/kg target delivered dose), respectively (Fig. 4f right panel). These results indicate that 5 mg/kg and 15 mg/kg doses in rats can respectively provide ~15-fold and ~94-fold higher local HH-120 concentration at 24 h post inhalation than the highest in vitro IC_90_ (1176 ng/mL) (Fig. 3b).

To evaluate whether HH-120 deposited in hamsters’ lungs can indeed exert viral neutralizing activity, we collected bronchoalveolar lavage fluid (BALF) from hamsters after inhalation and evaluated its neutralization activity against SARS-CoV-2 pseudotyped viruses (ancestral D614 strain). At different time points after HH-120 inhalation (15 min or 30 min, with HH-120 aerosols generated by nebulizing 5 mg/mL HH-120 solution), the neutralization activity of BALF collected at 12 h after 15 min or 30 min inhalation remained highly potent, achieving >90% neutralization of viral infection at a fourfold dilution (Fig. 4g). These results are consistent with the prediction based on in vitro IC_90_ values and PK results in rats and hamsters, further support that aerosolized HH-120 can be effectively deposited and exert neutralizing activity in the lungs of hamsters. Choosing a treatment regime of 15 min inhalation of HH-120 (nebulized from 5 mg/mL HH-120 solution) at a dosing frequency of twice daily (BID, every 12 h) should be suitable for in vivo efficacy studies.

### HH-120 inhalation shows potent efficacy in hamsters

In vivo efficacy of HH-120 was evaluated using a SARS-CoV-2 hamster infection model. The golden Syrian hamster is an appropriate animal model for testing the efficacy of vaccines or other anti-viral agents against SARS-CoV-2, as hamster ACE2 has been shown to bind to the S protein and to mediate SARS-CoV-2 entry^49^. Infection with SARS-CoV-2 in hamsters resembles mild-to-moderate disease of COVID-19^50^, or severe disease of COVID-19 when challenged with high virus doses^51^; however, infected hamsters typically recover after two weeks of infection^50^. Experimentally infected hamsters show significant weight loss and similar histopathological manifestations as human COVID-19 pneumonia^50,52^, displaying focal diffuse alveolar destruction, consolidation, inflammatory cells infiltration in the lungs; the infected hamster respiratory tract permits robust virus replication, with viral titers known to peak around 3 days after infection^52^.

**Fig. 5:**
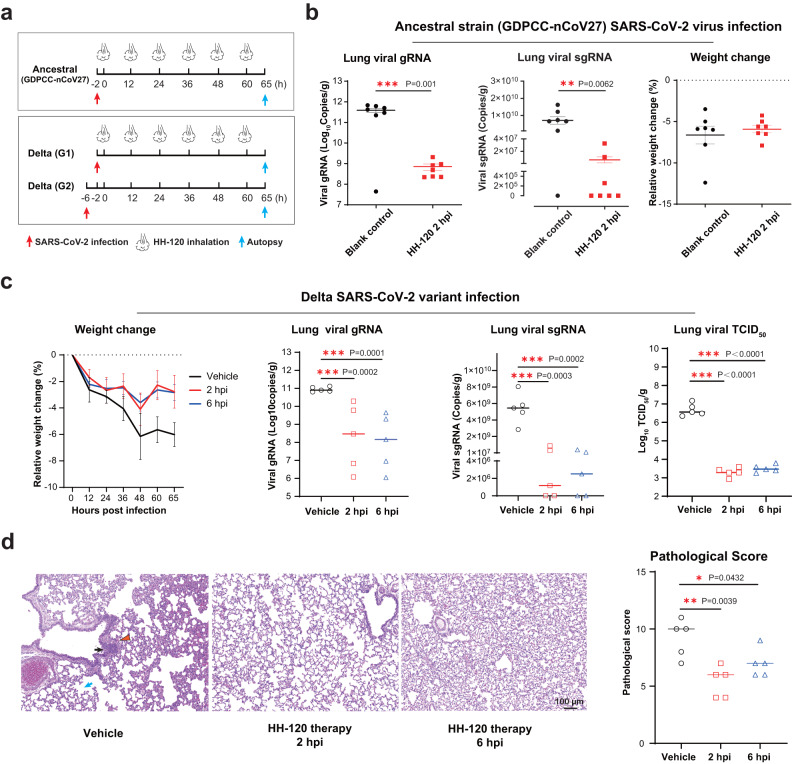
The therapeutic efficacy of inhaled HH-120 in treating hamsters infected with the SARS-CoV-2 ancestral strain and Delta variant. **a** Schematic illustration of the virus challenge experimental design. In ancestral strain (GDPCC-nCoV27) infection study, two groups of hamsters (*n* = 7/group) were challenged intranasally with 10^5^ pfu viruses. 2 h post viral challenge, one group of hamsters was given a theoretical delivered dose of ~0.5 mg/kg HH-120; the other group with no treatment served as a control group. In Delta variant infection study, three groups of male hamsters (*n* = 5/group) were infected with 10^4^ pfu of viruses. Two groups (2 h or 6 h post infection) of hamsters were given a theoretical delivered dose of ~2 mg/kg; the other group was given a placebo inhalation at 6 h post infection and served as a control group. **b** The therapeutic efficacy of inhaled HH-120 in treating hamsters infected with ancestral strain (GDPCC-nCoV27) SARS-CoV-2 (*n* = 7/group). At the end of treatment, hamsters were euthanatized, the lungs were collected, viral gRNA and sgRNA were measured by qRT-PCR. Relative weight change was assessed at the endpoint of the study. Horizontal lines represent mean values. **c** The therapeutic efficacy of inhaled HH-120 in treating hamsters infected with Delta SARS-CoV-2 (*n* = 5/group). The body weight was measured BID. Left lung lobes were collected after hamsters were euthanatized, viral gRNA and sgRNA were quantified by qRT-PCR, the TCID_50_ value was measured in a Vero cell infection experiment. **d** Pathology evaluation in Delta variant infection study. The right lungs were collected for H&E staining. Representative images of H&E staining are shown (left). Histopathological changes included mononuclear cells infiltration (blank arrow), hemorrhage (red triangle), widened alveolar septum (blue arrow). Pathological score (right) was obtained by evaluation of about 5 microscopic fields in one H&E staining slide. Error bars represent the SEM in panels (**b**) and (**c**) (Weight change). Horizontal lines represent median in panels (**c**) (gRNA, sgRNA, TCID_50_ viral titers) and (**d**) (pathological score). Statistical significance was analyzed by two-sided unpaired Student’s *t* tests (**b**–**d**). *p ≤ 0.05, **p ≤ 0.01, ***p ≤ 0.001. Source data are provided as a Source Data file.

For the SARS-CoV-2 ancestral strain (GDPCC-nCoV27) infection model, hamsters were challenged intranasally with 1  × 10^5^ pfu of SARS-CoV-2. HH-120 aerosol inhalation began at 2 h post infection, BID for 3 days (Fig. 5a). As described above, 15 min inhalation of HH-120 aerosols generated by nebulizing 5 mg/mL HH-120 solution was chosen as the inhalation dose. This dose resulted in a ~0.05 mg/kg lung deposited dose of HH-120 (Fig. 4d). After 3 days of BID treatment with HH-120 at 0.5 mg/kg delivered dose, hamsters were euthanatized, the anti-viral efficacy of HH-120 was evaluated by quantifying viral replication (gRNA and sgRNA) in the lungs using qRT-PCR. HH-120 significantly reduced the viral loads indicated by both gRNA and sgRNA in the lungs of all hamsters by about 3 orders of magnitude as compared to untreated hamsters (Fig. 5b), and sgRNA decreased to below the limit of detection in 4 of 7 hamsters. No significant weight change was observed in the treatment group (Fig. 5b).

For the SARS-CoV-2 Delta variant infection model, we increased the dose of HH-120 inhalation and lowered the challenge dose of the virus because Delta virus infection was more severe than that of ancestral strain (GDPCC-nCoV27) in hamster models, lowering the challenge dose can prevent premature death^53^. hamsters were challenged intranasally with 1 × 10^4^ pfu of the viruses. A fourfold higher delivered dose (~2 mg/kg) of HH-120 than that used in the ancestral strain (GDPCC-nCoV27) infection model was used in this experiment. This delivered dose was achieved by inhalation of HH-120 aerosols generated by nebulizing 10 mg/mL HH-120 solution for 15 min using two nebulizers in parallel. Buffer control aerosols generated in the same conditions were used as the vehicle group. HH-120 inhalation began at 2 h or 6 h post infection, and the treatment duration lasted for 3 days with a BID inhalation of 15 min each time (Fig. 5a). The weight of hamsters was measured BID. After 3 days of treatment, hamsters were euthanatized, the left lung lobes were collected for viral load analysis, viral gRNA and sgRNA were measured by qRT-PCR, and the TCID_50_ value was measured using Vero cell infection assays; the right lung lobes were collected for pathological analysis.

With HH-120 treatment, the infection-caused weight loss was clearly attenuated (Fig. 5c). Moreover, HH-120 inhalation significantly reduced the viral loads of gRNA and sgRNA in the hamster lungs by a median of about 3 orders of magnitude in both animal groups treated at 2 h or 6 h post viral challenge. The infectious viral titer (TCID_50_) in the lung lysates of HH-120 treatment group were also reduced by ~3 logs as compared to the vehicle group (Fig. 5c). The histopathological analysis showed that at 3 days post Delta variant infection, hamsters in vehicle group developed pulmonary damage, including hemorrhage, alveolar wall thickening, and infiltration of inflammatory cells in the lungs. HH-120 inhalation at both 2 h and 6 h post viral challenge attenuated pulmonary injury, with the pathological scores significantly lower than those of the vehicle group (Fig. 5d). These results demonstrate that HH-120 inhalation can greatly reduce viral loads in the lung and decrease viral infection-induced pathological damage when administered early after viral infection of the animals, warranting its further clinical development.

### HH-120 is safe in non-clinical toxicity studies

To evaluate the safety of HH-120 inhalation, we conducted repeat-dose inhalation toxicity studies in SD rats and cynomolgus monkeys following Good Laboratory Practice (GLP). In the SD rat study, HH-120 was given at target delivered dose levels of 5 and 15 mg/kg/dose BID, corresponding to actual delivered doses of 4.884 and 17.178 mg/kg/dose BID. In the monkey study, HH-120 was given at target delivered dose levels of 2.5 and 8 mg/kg/dose BID, corresponding to actual delivered doses of 2.831 and 9.202 mg/kg/dose BID. For both the rat and the monkey studies, HH-120 was given via an inhalation exposure system that was connected to Aerogen Solo nebulizer systems for two consecutive weeks, followed by a 2-week recovery period. Aerosol samples were collected simultaneously during the toxicity studies for aerosol particle size measurements using NGI. The MMAD range was determined to be 2.82–3.13 μm in the rat toxicity study and 3.38–3.60 μm in the monkey toxicity study. These particle size ranges were found to be appropriate for efficient delivery to both rat and monkey respiratory system^44,54^. All animals were evaluated for toxicological parameters, including clinical signs, vital functions body weight, food consumption, laboratory tests (hematology, coagulation, clinical chemistry parameters, urinalysis, cytokines, T lymphocyte subsets), organ weight, gross anatomy, and histopathology.

**Fig. 6:**
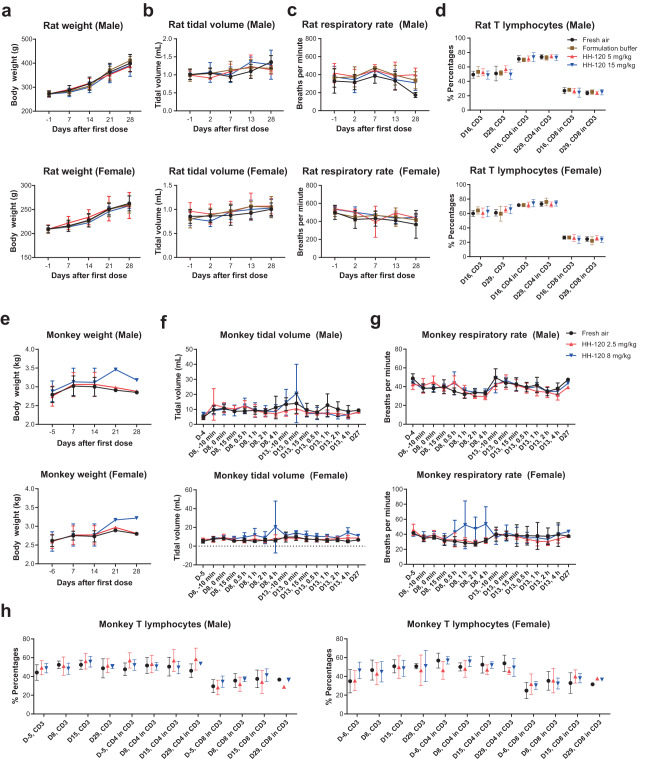
Safety evaluation of inhaled HH-120 in rats and monkeys. **a**–**d** Body weight (**a**), respiratory function (**b**–**c**), and T lymphocyte subset percentage changes (**d**) in two-week repeated dose toxicology study in rats. Four groups of SD rats (15/sex/group) were BID given fresh air, HH-120 formulation buffer or HH-120 at target delivered dose levels of 5 or 15 mg/kg/dose for 14 consecutive days followed by a two-week recovery period. 10/sex/group rats were euthanatized at the end of dosing period (D16) and 5/sex/group rats were euthanatized at the end of recovery period (D29). Tidal volume and respiratory frequency were detected by a rat WBP pulmonary system (**b**–**c**). Blood samples were collected on D16 and D29 from all euthanasia animals, CD3^+^, CD3^+^CD4^+^, CD3^+^CD8^+^ T lymphocyte subsets were analyzed by Flow Cytometry (**d**). **e**–**h** Body weight (**e**), respiratory function (**f**, **g**), and T lymphocyte subset changes (**h**) in two-week repeated dose toxicology study in monkeys. Three groups of cynomolgus monkey (5/sex/group) were BID given fresh air or HH-120 at target delivered dose levels of 2.5 or 8 mg/kg/dose for 14 consecutive days followed by a two-week recovery period. 3/sex/group rats were euthanatized at the end of dosing period (D15) and 2/sex/group rats were euthanatized at the end of recovery period (D29). Tidal volume and respiratory frequency were detected by a EMKA Non-invasive physiological signal telemetry system at the indicated timepoints post first dose at the indicated days (**f**, **g**). Blood samples were collected on five or six days before dose initiation, on D8, at the end of dosing (D15), and at the end of recovery period (D29), CD3^+^, CD3^+^CD4^+^, CD3^+^CD8^+^ T lymphocyte subsets were analyzed by Flow Cytometry (**h**). Data shown are the mean values (**a**–**h**). Error bars represent the SD (**a**–**h**). Source data are provided as a Source Data file.

HH-120 inhalation was well tolerated in both SD rats (Fig. 6a–d; Supplementary Tables 3–4) and cynomolgus monkeys (Fig. 6e–h; Supplementary Tables 3, 5) in the repeat-dose toxicity studies described above. In particular, HH-120 inhalation showed no adverse effects on body weight (Fig. 6a, e) and respiratory function (tidal volume and respiratory rate) for both rats and monkeys (Fig. 6b–c, f–g) during both the dosing and the recovery phases. No HH-120-realted adverse effects were observed on the percentage of CD3^+^, CD3^+^CD4^+^, and CD3^+^CD8^+^ T lymphocyte subsets (Fig. 6d, h), and serum levels of cytokines (TNF-α, IFN-γ, IL-2, IL-4 in rats and TNF-α, IFN-γ, IL-2, IL-4, IL-5, IL-6 in monkeys) for rats (only measured at the endpoints of dosing and recovery phases) and for monkeys (both dosing and recovery phases) (Supplementary Table 3).

In the rat toxicity study, the histopathological findings were minimal, sporadic, and unrelated to HH-120 (Supplementary Table 4). The no-observed-adverse-effect-level (NOAEL) was determined to be 17.178 mg/kg/dose (target delivered dose group of 15 mg/kg/dose) BID, which is ~30-fold higher than its effective delivered dose (~0.5 mg/kg/dose) tested in the hamster model.

In the monkey toxicity study, the major histopathological findings were limited to histological alterations in lungs: slight-to-moderate mononuclear cell infiltration in the perivascular region and interstitium of lungs in animals from both the 2.5 and the 8 mg/kg/dose groups, which was fully recovered by the end of two-week recovery period (Supplementary Table 5). Similar histological alterations in lungs were commonly observed in inhalation safety studies, which likely result from a local cleansing response mediated by immune systems^55^. Therefore, the observed mononuclear cell infiltration in lungs of animals from HH-120 dose groups was considered as a non-adverse effect, which is related to inhalation of macromolecule drug class rather than HH-120 specific. The NOAEL was determined to be 9.202 mg/kg/dose (target delivered dose group of 8 mg/kg/dose) BID. All these pre-clinical animal studies support a good safety profile for HH-120.

## Discussion

The ongoing emergence of SARS-CoV-2 variants has caused considerable concerns related to increased breakthrough infections after vaccination and escape from neutralizing antibody therapeutics^56^; it is therefore critical for public health efforts to continue the development of technologies to prevent and treat SARS-CoV-2 infections. It bears repeating that all known emerging SARS-CoV-2 viruses use hACE2 as the primary receptor, supporting the particular utility of developing hACE2-derived molecules as entry blockers that can confer broad-spectrum activities against SARS-CoV and SARS-CoV-2 infections^24,57^. The Omicron variant of SARS- CoV-2 offers an interesting case-in-point: this variant became more transmissible by increasing its affinity to hACE2^58,59^, so an hACE2-mimic recombinant protein that is potent and inherently resistant to viral escape would be assumed as a likely effective agent against the infection. However, a study reported that a bivalent hACE2-Fc fusion protein showed low neutralization activity to ancestral SARS-CoV-2 pseudotyped virus with an IC_50_ of 3.03 μg/mL^26^, and hACE2-hIgG1 (similarly a bivalent ACE2 with an Fc tag of hIgG1) in our study showed low activity as well in neutralizing authentic SARS-CoV-2 ancestral viruses, with an IC_50_ of 5.3 μg/mL. These findings together indicate that bivalent hACE2 lacks sufficient potency and therefore may has limited clinical application potential.

In the present study, we used an IgM-like design that enabled the gain of potency in neutralizing SARS-CoV-2 through increased avidity. Indeed, HH-120 confers about a hundred-fold higher neutralization activity than the Fc-tagged bivalent hACE2 (ACE2-hIgG1), which is comparable to EUA-issued monoclonal antibodies against SARS-CoV-2 variants prior to Omicron^60,61^. HH-120 is superior to most reported monoclonal antibodies in neutralizing the Omicron variant^3^ (with single-digit ng/mL IC_50_ values) in pseudotyped virus neutralization assays, although no side-by-side comparison has yet been conducted. Considering the broad-spectrum activity of HH-120 and its superior potency, it holds promising potential to be translated in humans as an effective agent for both prophylactics and early treatment of COVID-19.

A drug candidate named APN01, a soluble recombinant form of rhACE2, is currently under evaluation as a treatment (via infusion injection) for COVID-19. An earlier completed phase 2 trial (NCT04335136) with intravenously administered APN01 showed ambiguous efficacy in disease control for the treatment of hospitalized cases of COVID-19. A clinical trial for inhaled APN01 (NCT05065645) to assess safety, tolerability, pharmacokinetics, and pharmacodynamics was launched in Oct. 2021. Although the efficacy of APN01 against SARS-COV-2 remains to be tested, these clinical trials provide favorable safety evidence for use of ACE2-derived agents. ACE2 can catalyze the hydrolysis of angiotensin II into angiotensin and functions as a regulator in renin–angiotensin–aldosterone system (RAAS)^62^. Angiotensin II levels decreased rapidly following infusion of APN01, whereas angiotensin-(1–7) and angiotensin-(1–5) levels increased and remained elevated for 48 h in a phase 2 trial of APN01 (NCT01597635)^63^. Distinct from APN01, the enzymatic activity of hACE2 is deactivated in HH-120, eliminating the possibility of interfering with RAAS by the exogenous hACE2. Although a study indicates catalytic activity of ACE2 may slightly contribute to therapeutic efficacy for SARS-CoV-2 infection^64^, some ACE2 decoys are also designed to remove the catalytic activity for safety considerations^24,30^. Moreover, HH-120 does not contain the collectrin-like domain of hACE2, which mediates homodimerization of hACE2^21^. This simplifies protein engineering and purification of HH-120 and reduces the risk posed by soluble hACE2-mediated infection enhancement of SARS-CoV-2^65^.

Drug delivery through inhalation can be used for local and/or systemic action, depending on the ability of the aerosolized drug to cross the air-blood barrier. It is known that the diffusion of large therapeutics between the bloodstream and alveoli is limited, because the respiratory tract epithelium constitutes a barrier to the transport of large macromolecules (that do not diffuse passively through tight junctions)^14,66^. Given HH-120’s MW of nearly 1000 kDa, it was unsurprising when we observed no systemic drug exposure in rats or monkeys in our repeat-dose inhalation studies. Our detailed local PK profiling work in hamsters and rats and a comprehensive in vitro IC_90_ determination for various authentic viruses also help to provide basis for making extrapolations and predictions for dose selection and dosing frequency in future human studies. Beyond showing improved bioavailability of the drug and deceased potential for systemic side effects, our local delivery of aerosolized HH-120 to the respiratory tract in the COVID-19 context illustrates an attractive alternative administration route that is likely relevant to many future therapeutics.

In the present study, we demonstrated the local delivery of HH-120 by inhalation significantly reduced viral loads in the lung and decreased viral infection-induced pathological damage when administered early at 2 h or 6 h post-infection, supporting further clinical development of HH-120 for early treatment to prevent disease progression in humans. Nonetheless, the full therapeutic potential of HH-120 inhalation remains to be explored, testing at later timepoints post-infection (e.g. 12, 24 or 48 h) in animal models will help addressing whether HH-120 inhalation can be used for treating COVID-19 at a relatively late infection stage.

Since early 2022, the Omicron variant with high transmissibility and immune evasion ability has replaced Delta as the dominant variant. As the Omicron variant usually leads to upper respiratory system infection, it would be important to recalibrate the potential clinical benefit of the HH-120 inhalation in the Omicron era. Instead of reduction of hospitalizations or death from COVID-19, early use of HH-120 is likely more suitable for improving acute upper respiratory symptoms and post exposure prophylaxis through direct delivery to the upper respiratory tract (e.g. by nasal spray of the inhalable HH-120). Testing HH-120 being administered as soon as contracting or having had high-risk exposure to SARS-CoV-2 infection has been prioritized in clinical trials, a phase 2 (NCT05713318) clinical trial for early treatment and a phase 3 clinical trial (NCT05787418) for post-exposure prophylaxis are currently ongoing.

## Methods

### Protein expression

hACE2-hIgG1 was constructed by fusing the extracellular domain of human ACE2 (amino acid 19–615) (UniProt Q9BYF1) to the human IgG1 hinge and Fc (UniProt P01857). H374N and H378N mutations were introduced to inactivate the Zinc metallo-enzyme activity^17^. HH-120 was constructed by fusing hACE2-hIgG1 with an IgM tailpiece (PTLYNVSLVMSDTAGTCY)^33^. hACE2-hACE2-hIgG1 was constructed by tandemly fusing two hACE2 proteins to the N terminus of hIgG1. hACE2-hIgG1-hACE2 was constructed by fusing hACE2 to the N terminus and the C terminus of hIgG1. AbCR3022-hACE2 and Ab1-hACE2 were constructed by fusing hACE2 to the C terminus of CR3022 and Ab1. AbCR3022 is a monoclonal antibody with cross-binding activity to both SARS-CoV-2 and SARS-CoV^67^. Ab1 is a SARS-CoV-2 neutralization antibody developed in our lab. The coding sequences of the proteins were cloned into a pCAGGS vector or a pKS001 vector, and the proteins were produced in 293F cells (ThermoFisher)^68^ or CHO-K1Q cells (QuaCell) by either transient transfection or establishing stable cell lines. Cell cultures were harvested at a cell density of 1–2×10^7^/mL for next purification steps.

### SARS-CoV-2 RBD binding affinity and avidity analyses

SPR (BIAcore T200) and Bio-Layer Interferometry (BLI) (Fortebio Octet RED384) technologies were applied to measure the binding affinity and avidity of HH-120 and its bivalent form (hACE2-hIgG1) to SARS-CoV-2 RBD (amino acids 316-512 of the S protien in the SARS-CoV-2 D614 strain) or S trimer proteins of the Alpha, Beta, Delta, and Omicron variants (Sino Biological).

For affinity testing, the hACE2-hIgG1 or HH-120 was captured on the surface of a CM5 biosensor chip (Cytiva) coupled with an anti-human Fc antibody (Cytiva), twofold serially diluted SARS-CoV-2 RBD proteins or S trimer proteins of Alpha, Beta, Delta, Omicron variants (from 200 to 6.25 nM) in HBS-ET buffer were flowed through the chip at a speed of 30 μL/min, the binding constant (Ka), dissociation constant (Kd), and equilibrium dissociation constant (KD) were calculated using a 1:1 Langmuir binding model (BIA Evaluation software). For avidity testing, 20 μg/mL SARS-CoV-2 RBD proteins bearing His-avi tag were captured on the surface of a streptavidin biosensor (Sartorius, Octet), 2-fold serially diluted hACE2-hIgG1 (52.6–1.65 nM) or HH-120 (263.2–8.22 nM) were bound to the surface of the RBD-captured sensor for 180 s, and then dissociated for 300 s. A 1:1 binding model (Fortebio data Analysis 11.1-kinetics software) was used to calculate binding constants (Ka, Kd, and KD).

### Single-particle negative-stain EM

For negative-stain EM, purified HH-120 (3 μL of 0.12 mg/mL) was applied to a glow-discharged carbon coated copper grid for 45 s and was then blotted off with filter paper. The grid was washed using washing buffer (25 mmol/L Tris-HCl, 100 mmol/L Arg-HCl, pH7.5), and then immediately stained using 2% uranyl acetate solution. Data were acquired on a Talos L120C electron microscope operated at 120 kV equipped with an FEI Ceta 4 K detector. Images were collected at a pixel size of 3.122. Data processing including micrograph screening, particle picking, particle selection. 2D reconstruction were performed using CryoSparc v3.3. Patch CTF estimation was used to check the defocus (−1 to −3 μm) and max resolution of all micrographs. Several rounds of 2D classifications were performed to remove poor-quality particles and about 25,343 high-quality particles were picked for final HH-120 2D model construction.

### ADCC and ADCP reporter assays

Jurkat/NFAT-luc2p/FcγRIIa, Jurkat/NFAT-luc2p/ FcγRIIIa cell lines, CHO cells stably expressing SARS-CoV-2 S protein (CHO-S) established in-house were employed as effector cells and target cells, respectively. CHO-S cells (15,000 cells/well) were seeded in a white, flat bottom, opaque 96-well plate and cultured for 16–20 h at 37 °C, followed by brief incubation with serially diluted HH-120. The effector cells were then added at an effector-to-target cell ratio (E:T) of 6:1. Luciferase activities were measured using a Bright-Glo^TM^ Luciferase Assay System (Promega) and a Centro LB 960 Microplate Luminometer (Berthold).

### Pseudovirus neutralization assay

Plasmids encoding S proteins of Pangolin-CoV, SARS-CoV, SARS-CoV-2 D614, G614, G614 variants bearing single-site mutations (L18F, A222V, V367F, K417N, N439K, L452R, Y453F, S477N, S477I, T478A, T478I, E484K, F486L, N501Y, A520S, P1263L), Alpha (B.1.1.7), Beta (B.1.351), Epsilon (B.1.427/429), Gamma (P.1), Kappa (B.1.617.1), Delta (B.1.617.2), and Omicron (B.1.1.529, BA.1.1, BA.2, BA2.12.1, BA.4 or BA.5, BA.2.75, BA.2.76, BF.7, XBB, BQ.1.1) variants were constructed respectively. SARS-CoV-2 pseudotyped viruses were produced using a lentivirus system^69^ (prepared in house) or a VSV system^70^ (Vazyme #DD1201). For lentivirus system based pseudotyped virus preparation, 293 T cells were co-transfected with S protein expression plasmid, a lentiviral packaging plasmid, and a plasmid harboring a firefly luciferase (FLuc) reporter gene (1:3:4). Viral supernatants were collected 48 h after transfection. Viral titers were measured as luciferase activity (using relative light units; Bright-Glo Luciferase Assay System).

Neutralization assays were performed using the 293T-ACE2 stable cell line. Pseudotyped viruses were incubated with serially diluted HH-120 at room temperature for 30 min. The mixtures were then added to 96-well plates that had been seeded with 1.0 × 10^5^ (lentivirus system) or 8.0 × 10^4^ (VSV system) 293T-ACE2 cells/well one day prior to infection. After a 24 h incubation, the cells were washed and further incubated for 24 h. Luciferase activity was measured on a Centro LB 960 Microplate Luminometer (Berthold) or an EnVision 2105 Multimode Plate Reader (PerkinElmer) using a Bright-Glo Luciferase Assay System (Promega). The percentages of infectivity were calculated as the ratio of luciferase readout in the presence of HH-120 normalized to the luciferase readout of PBS controls. The IC_50_ values were determined using 4-parameter logistic regression (GraphPad Prism). Cells without viral infection were used as blank controls.

### Authentic live virus neutralization assay

Neutralization assays were performed under biosafety level-3 (BSL-3) conditions. Neutralization activity of HH-120 or hACE2-hIgG1 against SARS-CoV-2 prototype strain (EPI_ISL_402119) was analyzed using Vero cells as host cells, and the assay was performed as described previously^71^ at the National Institute for Viral Disease Control and Prevention, China CDC. Briefly, Vero cells were seeded in a 96-well plate with 2.0 × 10^4^ cells/well and incubated at 37 °C one day prior to virus infection. Viruses at titer of 200 TCID_50_ were mixed with serial diluted HH-120 or hACE2-hIgG1 respectively, incubated at 37 °C for 1 h, added the mixture to Vero cells. After 48 h incubation, Cytopathic effect (CPE) was observed, and 100 μL of the culture supernatant was harvested for qRT-PCR analysis. TCID_50_ of the virus in the sample was calculated according to the CT value of the sample and a standard curve. The neutralization potency was calculated as follows: Inhibition % = (TCID_50_ without drug—TCID_50_ with drug)/ TCID_50_ without drug ×100%. The ancestral strain (GISAID accession: EPI_ISL_402119) was isolated from a SARS-CoV-2 infected patient by China CDC.

HH-120’s neutralization activity against SARS-CoV-2 ancestral strain (GDPCC-nCoV27), Alpha, Beta, Delta, Omicron (XBB.1.16) variants was analyzed using Vero cells as host cells, and the assay was performed as described previously^72^ at National Kunming High-level Biosafety Primate Research Center, Yunnan. Briefly, Vero host cells (4.0 × 10^4^ cells/well) were seeded in a 96-well plate and incubated at 37 °C one day prior to virus infection. Then, the Vero cells were pre-treated with different doses of HH-120 for 1 h, and the virus (multiplicity of infection (MOI) = 0.05) was subsequently added to allow infection for 1 h. At 48 h post infection, CPEs were observed and viral yield in the cell supernatant was then quantified by qRT-PCR. The ancestral strain (GDPCC-nCoV27) was from Guangdong CDC, China.

HH-120’s neutralization activity against SARS-CoV-2 Omicron variant (B.1.1.529) was analyzed using HK-2 cells as host cells, and the assay was performed as described previously^73^ at the Carol Yu Centre for Infection of the University of Hong Kong. Briefly, HH-120 was serially diluted in 5-fold starting from 10 μg/mL. Duplicates of each sample were mixed with 100 TCID_50_ of Omicron variant (B.1.1.529) for 1 h, and the HH-120 virus mixture was then added to HK-2 cells. After incubation for 3 days, CPE was examined. For all the authentic virus neutralization assays, the IC_50_ values were determined using 4-parameter logistic regression. Cells without viral infection were used as blank controls.

### SARS-CoV-2 S protein mediated syncytia formation assay

293T cells co-transfected with plasmids expressing the SARS-CoV-2 S protein and pEGFP-N1 were termed “S cells”, while 293T cells co-transfected with plasmids expressing hACE2-C9 and pmCherry-C1 were termed “ACE2 cells”. 24 h after transfection, both the S cells and ACE2 cells were digested with EDTA and washed once with DMEM complete medium. The S cells were then pre-incubated with the HH-120 or hACE2-hIgG1 at 37 °C for 30 min before being co-cultured with the ACE2 cells at a cell ratio of 1:1. After another 3 h or 20 h of co-culture, S-ACE2 interaction-mediated syncytia formation (assessed as the mergers of green and red cells) was imaged under a fluorescence microscope (Eclipse Ti, NIKON). 293T cells transfected with the EGFP plasmid alone co-culturing with ACE2 cells were used as a mock control. DMEM completed medium without drug were used as a negative control (blank).

### Aerodynamic assessment of HH-120 nebulized using a mesh vibrating nebulizer

APSD profiles were measured by an NGI (Model 170, COPLEY) in accordance with the instructions. Briefly, an NGI was connected to a flow meter and a vacuum source generating a 15 L/min inlet flow rate. The NGI inlet nozzle was connected to an Aerogen Solo nebulizer through an adaptor. The cut-off diameters for the stage 1 to stage 7 of NGI at volumetric flow rate of 15 L/min were 14.1, 8.61, 5.39, 3.30, 2.08, 1.36, and 0.98 μm, respectively. HH-120 aerosols were generated by nebulizing 10 mg/mL HH-120 solution for 8 min. The average nebulized volume was about 2 mL in 6 independent experiments. HH-120 deposited on the nebulizer, mouthpiece adaptor (MA), induction port (IP), and 7 stages of NGI was washed off by HH-120 formulation buffer. Collected HH-120 was quantified by SEC-HPLC. FPF%, MMAD, GSD were determined using Copley Inhaler Testing Data Analysis Software (CITDAS). Drug delivery rate and total drug delivered were measured by breathing simulator (BRS1100, COPLEY) in accordance with the instructions. Briefly, breathing simulator was set at the adult pattern with tidal volume of 500 mL, breath frequency of 15 times/min, sinusoidal waveform, Inhalation/Exhalation (I:E) Ratio of 1:1. An Aerogen Solo nebulizer was connected to a breathing simulator through an adaptor. HH-120 aerosols were generated by nebulizing 5 mL of 10 mg/mL HH-120 solution. HH-120 deposited on the filter membrane (COPLEY) which reflects total drug delivered was washed off by blank solvent and quantified by SEC-HPLC. The delivery rate was calculated as total drug delivered divided by nebulization time.

### Respiratory tract deposition assay in hamsters via aerosol inhalation

The animal studies were reviewed and approved by the Institutional Animal Care and Use Committee of the National Institute of Biological Sciences, Beijing. Male or female (6–8 w of age), specific-pathogen-free golden Syrian hamsters (Hebei Invivo Biotech Co. Ltd.) were used. HH-120 inhalation was delivered to hamsters using a small animal whole-body inhalation exposure system customized by Yuyan Instruments Inc., which is comprised an air pump, a mass flowmeter, and an exposure box. The exposure box, which can accommodate at least 8 hamsters, can be interfaced with the adapter of a Aerogen Solo nebulizer. HH-120 aerosols were generated by nebulizing 5 mg/mL HH-120 solution using the Aerogen Solo nebulizer. For hamster respiratory tract deposition profile characterization, immediately after 50 min of HH-120 inhalation, two hamsters per group were euthanized, NLF was collected through entire nasal cavity lavage with 0.9% saline, and the trachea, bronchus, and lung tissues were harvested and digested in RIPA lysis buffer. For testing of the lung PK profile of inhaled HH-120, three groups of hamsters (*n* = 6/group) were given a single dose (5-min, 15-min, or 50-min) inhalation of HH-120 aerosols. At different time points (0, 6 and 24 h) after the end of inhalation, two hamsters of each group at each time point were euthanized and lungs were collected. For neutralization activity analysis of BALF, two groups of hamsters (*n* = 4/group) were given a single dose (15-min or 30-min) inhalation of HH-120 aerosols. Immediately (0 h) and 12 h after inhalation, two hamsters of each group at each time point were euthanized and BALF was collected from the euthanized hamsters. Serially diluted BALF samples were then analyzed for neutralization activities against pseudotyped SARS-CoV-2 viruses (D614). The amount of HH-120 in serum, lavage, and lysate were determined using RBD capture ELISA with a standard HH-120 sample.

### PK evaluation of inhaled HH-120 in SD rats

The study was reviewed and approved by the Institutional Animal Care and Use Committee of the Institute of JOINN Laboratories, China. Male or female, specific-pathogen-free SD rat (6–8 w of age) (Zhejiang Vital River Laboratory Animal Technology Co., Ltd.) were used. HH-120 aerosols were generated by nebulizing 10 mg/mL HH-120 solution and were given via an inhalation exposure system connected to four Aerogen Solo nebulizer systems. The delivered dose was calculated using the formula: Delivered dose (mg/kg) = RMV × Ec × T/BW^74^. RMV (L/min) = Respiratory Minute Volume = 0.608 × BW (kg)^0.852^; Ec (mg/L air) = Concentration delivered to the animals; T (min) = Duration of exposure; BW (kg) = Body weight. Before dosing, the target delivered dose was calculated using the above formula with Ec = Estimated delivery concentration deduced from previous tests for measuring aerosolized HH-120 convention by filter sampling method; after dosing, the actual delivered dose was calculated using the same formula with Ec = actual concentration directly measured by filter sampling during the PK study. A total of 58 SD rats were randomly assigned to two groups (14–15/sex/group) and given HH-120 at target-delivered doses of 5 or 15 mg/kg/dose. At 0 (<5 min), 6, 12, 24, and 48 h after completion of a single dose of HH-120 inhalation (*n* = 3/sex except *n* = 2–3/sex for 48 h timepoint), rats were euthanized and blood serum samples, nasal lavage fluid (NLF), and larynx, trachea, and lung tissues of the rats were collected separately. The amount of HH-120 in serum, lavage, and tissues lysates were measured using RBD capture ELISA.

### RBD capture ELISA

2 μg/mL biotin-labeled COVID-19 RBDs were captured on a 96-well plate that was pre-coated with 2 μg/mL streptavidin. Purified HH-120 or diluted samples (NLF, BALF, or tissue lysate supernatants) were added to the plate; HRP-labeled goat anti-hFc secondary antibody (Thermo Fisher, Pierce) was used as the detection antibody. The optical density (OD) was measured at OD_450—_OD_630_ (BioTek).

### SEC-HPLC

Purified HH-120, hACE2-hIgG1, or HH-120 washed off in the aerosol assessment assays were analyzed via SEC-HPLC using a TSKgel G4000SWxl analytical column (Tosoh) with an Agilent 1260 HPLC system. 100 μg of HH-120 or hACE2-hIgG1 (in 50 mM PB, 300 mM NaCl at pH 6.7 ± 0.1 buffer) was injected to the system at a flow rate of 0.8 mL/min.

### SEC-MALS

The system consisted of an Agilent 1200 HPLC system, a DAWN MALS detector (Wyatt Technology), an Optilab differential Refractive Index (dRI) detector (Wyatt Technology), and a TSKgel G4000SWxl analytical column (Tosoh). PBS was used as the running buffer with a flow rate of 0.8 mL/min. Data acquisition was performed using Astra software (Wyatt Technology).

### SV-AUC

SV-AUC experiments were carried out using a 12-mm charcoal-filled Epon centerpieces (Beckman, 392778) and a four-hole An60 Ti rotor at 30,000 rpm (Optima AUC analytical-ultracentrifuge, Beckman Coulter) with absorbance detection at 280 nm at 20 °C. The data were analyzed with SEDFIT software to obtain sedimentation coefficient distribution C (S)^75^.

### Hamster efficacy study of HH-120 inhalation

The animal studies were reviewed and approved by the Institutional Animal Care and Use Committee of the Institute of Medical Biology of the Chinese Academy of Medical Science and were performed in the animal biosafety level 3/4 (ABSL-3/4) facility of the National Kunming High-level Biosafety Primate Research Center, Yunnan, China. Male (6–8 w of age), specific-pathogen-free golden Syrian hamsters (Hebei Invivo Biotech Co. Ltd.) were used. All hamsters were provided access to standard pelleted feed and water ad libitum for 1 week of acclimatization after arrival. Prior to being moved into the ABSL facility for experimental challenge, hamsters were housed individually in isolated ventilation cages.

For the SARS-CoV-2 ancestral strain (GDPCC-nCoV27) infection model, hamsters were infected with 10^5^ pfu of the viruses intranasally and subsequently exposed for 15 min of HH-120 aerosols generated by nebulizing 5 mg/mL HH-120 solution using the Aerogen Solo nebulizer at 2 h post-infection. Animals were assigned to two groups: control group with no aerosol inhalation treatment (*n* = 7), and 15 min exposure to HH-120 aerosols BID (*n* = 7), for a total of 6 exposures.

For the SARS-CoV-2 Delta variant infection model, hamsters were challenged intranasally with 10^4^ pfu of the viruses and subsequently exposed for 15 min of HH-120 formulation buffer (placebo control) or HH-120 aerosols generated by nebulizing 10 mg/mL HH-120 solution using two Aerogen Solo nebulizers in parallel at 2 h or 6 h post-infection (*n* = 5) for a total of 6 exposures.

Baseline body weights were measured for all hamsters before viral challenges and monitored BID after HH-120 inhalation. The hamsters were monitored BID for signs of COVID-19 disease (ruffled fur, hunched posture, labored breathing, anorexia, lethargy) post-challenge with SARS-CoV-2. At necropsy, the left lung lobes were collected for viral load quantification. In the Delta variant infection experiment, at the endpoint, the right lung lobes were collected for H&E staining.

Histopathological examination using H&E staining method was performed as described previously^76^. Briefly, the histopathological changes of lung, such as inflammation, structure change and hemorrhage, were graded according to the following scoring system. Score 0 indicates clear structure of alveolar without inflammatory infiltration. Score 1 indicates mild inflammation, slightly widened alveolar septum and sparse mononuclear cells (monocytes and lymphocyte) infiltration. Score 2 indicates severe inflammation, thickening of alveolar wall and increased inflammatory infiltration of interstitial monocytes. Score 3–4 indicates alveolar septum widened significantly, increased infiltration of inflammatory cells. Score 5 indicates extensive exudation and widened septum, smaller alveolar cavity, septal bleeding, and infiltration of alveolar cells. Score >5 indicates a large number of cells infiltrated into the alveolar cavity; the alveolar cavity disappeared; the septum fused, and a transparent membrane was formed on the alveolar wall. Five fields of each section were randomly chosen for evaluation of histopathological changes according to the scoring system above. Histopathological changes of the lung from every hamster were graded based on thickening or consolidation of pulmonary septum, bleeding of pulmonary septum, infiltration of inflammatory cells, vascular thrombosis and distribution area of dust cells. The total score of each index is the final score of histopathological changes for each hamster.

qRT-PCR (Bio-Rad, CFX384) was used to measure viral gRNA and sgRNA (the latter of which is indicative of virus replication) in lung tissues. Data are normalized by tissue weight and are reported as copies of RNA determined by comparing the cycle threshold (CT) values from the unknown samples to CT values from a positive-sense SARS-CoV-2 RNA standard curve^77^. The primers and probe sequences used for gRNA were derived from the N gene (forward, 5′-GGGGAACTTCTCCTGCTAGAAT-3′; reverse, 5′-CAGACATTTTGCTCTCAAGCTG-3′; probe, 5′-FAM-TTGCTGCTGCTTGACAGATT-TAMRA-3′), according to the sequences recommended by the WHO and the China CDC. SARS-CoV-2 E gene subgenomic mRNA (sgRNA), indicative of virus replication, was assessed by qRT-PCR as previously described^78^, using the following primers and probe sequences (forward, 5′-CGATCTCTTGTAGATCTGTTCTC-3′; reverse, 5′-ATATTGCAGCAGTACGCACACA-3′; probe, 5′-FAM-ACACTAGCCATCCTTACTGCGCTTCG-BHQ-3′). TCID_50_ was measured using Vero cell infection experiment.

### Toxicology studies in SD rats and monkeys

All toxicology studies followed GLP and were reviewed and approved by the Institutional Animal Care and Use Committee of the Institute of JOINN Laboratories, China. Male and female (6–8 w of age), specific-pathogen-free SD rat (Zhejiang Vital River Laboratory Animal Technology Co., Ltd.), as well as male and female (2.9–3.7 years of age) cynomolgus monkeys (Guangxi Weimei Bio-Tech CO., Ltd.) were used. For both the rat and the monkey studies, HH-120 aerosols were generated by nebulizing 10 mg/mL HH-120 solution and were given via an inhalation exposure system connected to four Aerogen Solo nebulizer systems. The target-delivered doses and the actual delivered doses were calculated using the same method as for the rat PK study. The first dosing day was defined as Day 1.

Two-week repeated dose toxicology study in SD rats: A total of 120 SD rats (60/sex) were randomly assigned to four groups (15/sex/group), and given fresh air, HH-120 formulation buffer, or HH-120 BID at target delivered dose levels of 5 or 15 mg/kg/dose (corresponding to actual delivered doses of 4.884 or 17.178 mg/kg/dose) for 14 consecutive days followed by a two-week recovery period. 10/sex/group rats were euthanatized at the end of dosing period (D16) and 5/sex/group rats were euthanatized at the end of recovery period (D29). Toxicological parameters evaluated included clinical observations, ophthalmology, respiratory functions, body weight, food consumption, laboratory tests (hematology, coagulation, clinical chemistry, urinalysis, cytokines, T lymphocyte subsets), organ weight, gross anatomy, and histopathology. Respiratory functions (tidal volume and respiratory frequency) were monitored with a EMKA whole body plethysmography (WBP) pulmonary system (EMKA TECHNOLOGIES Inc.) before dose initiation (D-1), within 2 h after the first dose on D2, D7, D13, and D28 (the recovery period). Data were analyzed by a IOX software (Version 2.9.4.31). Blood samples were collected on D16 and D29 from all euthanasia animals, CD3^+^, CD3^+^CD4^+^, CD3^+^CD8^+^ T lymphocyte subsets were analyzed by Flow Cytometry (BD LSRFortessa) (Supplementary Fig. 5a); TNF-α, IFN-γ, IL-2 and IL-4 levels in serum were measured using Cytometric Bead Array (CBA) kits by Flow Cytometry (BD LSRFortessa).

Two-week repeated dose toxicology study in monkeys: A total of 30 cynomolgus monkeys (15/sex) were randomly assigned to three groups (5/sex/group) and given fresh air or HH-120 BID at target delivered dose levels of 2.5 or 8 mg/kg/dose (corresponding to actual delivered doses of 2.831 or 9.202 mg/kg/dose) for 14 consecutive days followed by a 2-week recovery period. At the end of dosing period (D15), 3/sex/group monkeys were euthanatized, and remaining monkeys (2/sex/group) were euthanatized at the end of recovery period (D29). Toxicological parameters evaluated included clinical observations, ophthalmology, ECG waveforms, blood pressure, body temperature, respiratory functions, oxygen saturation, body weight, food consumption, laboratory tests (hematology, coagulation, clinical chemistry, urinalysis, cytokines, C3 and C4, T lymphocyte subsets), organ weight, gross anatomy, and histopathology. Respiratory functions (tidal volume and respiratory frequency) were monitored with a EMKA non-invasive physiological signal telemetry system before dose initiation (D-5), 10 min before the first dose on D8 and immediately (<10 min), 0.25 h, 0.5 h, 1 h, 2 h, 4 h post the first dose on D8, D13 and at the recovery period (D27). Data were analyzed by IOX software (Version 2.10.8.6). Blood samples were collected on five or six days before dose initiation, on D8, at the end of the dosing (D15), at the end of the recovery period (D29), CD3^+^, CD3^+^CD4^+^, CD3^+^CD8^+^ T lymphocyte subsets were analyzed by Flow Cytometry (BD FACSCalibur) (Supplementary Figure 5b); TNF-α, IFN-γ, IL-2, IL-4, IL-5, and IL-6 levels in serum were measured using CBA kits by Flow Cytometry (BD FACSCalibur).

At the end of dosing period and recovery period, animals were humanly euthanized followed by complete necropsy. Tissues, including adrenal glands, aorta, bone with bone marrow from femur and sternum, brain, nasal cavity (turbinate, nasopharynx and nasal associated lymphoid tissue), epididymides, esophagus, eyes with optic nerves, heart, kidneys, lacrimal glands, large intestine (cecum, colon, rectum), larynx, liver, lung with bronchi, lymph nodes (bronchial, mandibular, mesenteric, inguinal), mammary gland, sciatic nerve, ovaries with oviducts, peyer’s Patch, pancreas, prostate, pituitary, salivary glands (parotid, mandibular, sublingual), seminal vesicles, skeletal muscle (biceps femoris), skin (close to mammary), small intestines (duodenum, jejunum, ileum), spinal cord (cervical, thoracic, lumbar), spleen, stomach, testes, thymus, thyroid glands with parathyroids, tongue, trachea, urinary bladder, uterus with cervix, and vagina tissues in both rats and monkeys; harderian glands in rats; gallbladder in monkeys were collected. Collected tissues were processed with routine histological methods: embedded in paraffin, sectioned, mounted on slides, and stained with hematoxylin and eosin (H&E). Slides from fresh air group and 15 mg/kg HH-120 group in the rat study and all tissue slides in monkey studies were examined microscopically by a certified pathologist and peer reviewed by a JSTP (Japanese Society of Toxicologic Pathology) board certified pathologist. Findings were described and categorized by the study pathologist using standardized nomenclature^79,80^. A five-step grading system of minimal, slight, moderate, marked, or severe was used to rank the severity of microscopic lesions for comparison among groups as appropriate (Supplementary Table 6).

### Statistics

All statistical analyses were performed using GraphPad Prism 9 and the statistic tests are described in the figure legends.

### Supplementary information

Supplementary Tables and Figures

## Data Availability

All data are included in the article and supplementary materials. Source data are provided with this paper.
