# Supplementary material for: An IgM-like inhalable ACE2 fusion protein broadly neutralizes SARS-CoV-2 variants
Source: Nat Commun. 2023 Aug 25;14:5191. doi: 10.1038/s41467-023-40933-3 (PMC10457309; doi:10.1038/s41467-023-40933-3)
Supplement: Supplementary file 1 — Supplementary Tables and Figures [file 41467_2023_40933_MOESM1_ESM.pdf]

Supplementary Table 1

a

Neutralization activities of HH-120 aganist 16 pseudotyped SARS-CoV-2 G614 variants each containing a single mutation in the Spike protein

Mutations	L18F	A222V	V367F	P1263L	K417N	N439K	L452R	Y453F	S477N	S477I	T478A	T478I	E484K	F486L	N501Y	A520S
IC_{50} (ng/mL)	2.89	1.15	1.98	1.84	0.49	0.46	0.59	0.83	0.55	0.58	2.09	1.88	0.38	0.60	0.49	0.63
IC_{90} (ng/mL)	26.01	10.35	17.82	16.56	4.41	4.14	5.31	7.47	4.95	5.22	18.81	16.92	3.42	5.40	4.41	5.67

b

Neutralization activities of HH-120 aganist pseudotyped SARS-CoV-2 variants

WHO label	Pango Lineage	Mutations in Spike protein	Pseudotyped virus system	IC_{50} (ng/mL)	IC_{90} (ng/mL)
Alpha	B.1.1.7	del69-70/del144-145/N501Y/A570D/D614G/P681H/T716I/S982A/D1118H	Lentivirus system	0.59	5.31
Beta	B.1.351	K417N/E484K/N501Y/D614G		2.59	23.31
		D80A/D215G/K417N/E484K/N501Y/D614G/A701V		2.73	24.57
		D80A/D215G/del241-243/K417N/E484K/N501Y/D614G/A701V		0.58	5.22
Gamma	P.1	K417T/E484K/N501Y/D614G		0.24	2.16
Delta	B.1.617.2	T19R/G142D/del157-158/L452R/T478K/D614G/P681R/D950N		0.41	3.69
		T19R/DEL157/158/L452R/T478K/D614G/P681R/D950N		0.55	4.95
Epsilon	B.1.427/429	S13I/W152C/L452R/D614G		0.76	6.84
Kappa	B.1.617.1	L452R/D614G/P681R		2.21	19.80
		L452R/E484Q/D614G/P681R		1.16	10.44
Omicron	B.1.1.529	A67V/del69-70/T95I/G142D/del143-145/del211/L212I/ins214EPE/G339D/S371L/S373P/S375F/K417N/N440K/G446S/S477N/T478K/E484A/Q493K/G496S/Q498R/N501Y/Y505H/T547K/D614G/H655Y/N679K/P681H/N764K/D796Y/N856K/Q954H/N969K/L981F	VSV system	3.93	35.37
	BA.1.1	A67V/del69-70/T95I/G142D/del143-145/del211/L212I/ins214EPE/G339D/R346K/S371L/S373P/S375F/K417N/N440K/G446S/S477N/T478K/E484A/Q493K/G496S/Q498R/N501Y/Y505H/T547K/D614G/H655Y/N679K/P681H/N764K/D796Y/N856K/Q954H/N969K/L981F		4.29	38.61
	BA.2	T19I/L24S/ del25-27/ G142D/ V213G/ G339D/S371F/S373P/ S375F/ T376A/ D405N/ R408S/ K417N/ N440K/ S477N/ T478K/ E484A/ Q493R/ Q498R/ N501Y/ Y505H/ D614G/ H655Y/ N679K/ P681H/ N764K/ D796Y/ Q954H/N969K		5.30	47.70
	BA.2.12.1	T19I/L24S/ del25-27/ G142D/ V213G/ G339D/S371F/S373P/ S375F/ T376A/ D405N/ R408S/ K417N/ N440K/L452Q/ S477N/ T478K/ E484A/ Q493R/ Q498R/ N501Y/ Y505H/ D614G/ H655Y/ N679K/ P681H/S704L N764K/ D796Y/ Q954H/ N969K		4.62	41.58
	BA.4 or BA.5	T19I/L24S/ del25-27/del69-70/G142D/ V213G/ G339D/S371F/S373P/ S375F/ T376A/ D405N/ R408S/ K417N/ N440K/L452R/ S477N/ T478K/ E484A/F486V/ Q498R/ N501Y/ Y505H/ D614G/ H655Y/ N679K/ P681H/ N764K/ D796Y/ Q954H/ N969K		3.74	33.66

Supplementary Table 2

Detailed aerosol particle size distribution in different size ranges determined by NGI

	MA + IP	Stage							MOC
		1	2	3	4	5	6	7	
**Particle diameter range (μm)**	**–**	**> 14.10**	**8.61–14.10**	**5.39–8.61**	**3.30–5.39**	**2.08–3.30**	**1.36–2.08**	**0.98–1.36**	**<0.98**
**Percentage (%)**	**2.53**	**7.94**	**13.27**	**18.60**	**27.42**	**19.57**	**6.77**	**2.44**	**1.46**

Supplementary Table 2. Detailed aerosol particle size distribution profiles of HH-120 after inhalation measured by NGI. HH-120 aerosols were generated by nebulizing 10 mg/mL HH-120 solution for 8 min. The cut-off diameters for the stage 1 to stage 7 of NGI at volumetric flow rate of 15 L/min were 14.1 μm, 8.61 μm, 5.39 μm, 3.30 μm, 2.08 μm, 1.36 μm, and 0.98 μm, respectively. The average nebulized volume was about 2 mL in 6 independent experiments. HH-120 deposited on the nebulizer, mouthpiece adaptor (MA), induction port (IP), 7 stages of NGI, and Micro-orifice contact (MOC) were collected and quantified by SEC-HPLC.

Supplementary Table 3

a

Serum levels of cytokines in the rat toxicity study

Cytokines[1]	Days	Fresh air		Formulation buffer		HH-120 5 mg/kg		HH-120 15 mg/kg	
		Mean (pg/mL)	Animal numbers for calculation[2]	Mean (pg/mL)	Animal numbers for calculation	Mean (pg/mL)	Animal numbers for calculation	Mean (pg/mL)	Animal numbers for calculation
Male (n=10/group at D16, n=5/group at D29)									
IL-2	D16	NC[3]	0	NC	0	6.17	1	11.70	2
	D29	NC	0	NC	0	NC	0	NC	0
IL-4	D16	10.01	1	4.82	1	NC	0	46.50	3
	D29	8.25	1	7.72	2	6.87	2	6.71	1
TNF-α	D16	20.54	2	34.22	3	201.96	6	444.78	3
	D29	NC	0	13.70	1	18.07	2	NC	0
IFN-γ	D16	6.37	2	3.35	1	13.30	3	NC	0
	D29	3.85	2	NC	0	NC	0	NC	0
Female (n=10/group at D16, n=5/group at D29)									
IL-2	D16	13.46	2	NC	0	NC	0	NC	0
	D29	NC	0	NC	0	NC	0	NC	0
IL-4	D16	5.59	1	NC	0	NC	0	NC	0
	D29	4.24	3	12.41	3	1.81	2	4.59	1
TNF-α	D16	55.75	3	NC	0	34.65	1	NC	0
	D29	131.05	1	79.27	1	NC	0	NC	0
IFN-γ	D16	8.66	2	NC	0	16.37	1	NC	0
	D29	NC	0	NC	0	NC	0	NC	0

b

Serum levels of cytokines in the monkey toxicity study

Cytokines	Days	Fresh air		HH-120 2.5 mg/kg		HH-120 8 mg/kg		Days	Fresh air		HH-120 2.5 mg/kg		HH-120 8 mg/kg	
		Mean (pg/mL)	Animal numbers. for calculation	Mean (pg/mL)	Animal numbers for calculation	Mean (pg/mL)	Animal numbers for calculation		Mean (pg/mL)	Animal numbers for calculation	Mean (pg/mL)	Animal numbers for calculation	Mean (pg/mL)	Animal numbers for calculation
Male (n=5/group at D-5, D8, D15; n=2/group at D29)								Female (n=5/group at D-5, D8, D15; n=2/group at D29)						
IL-2	D-5	NC	0	NC	0	NC	0	D-6	NC	0	NC	0	NC	0
	D8	NC	0	NC	0	NC	0	D8	NC	0	NC	0	NC	0
	D15	NC	0	NC	0	NC	0	D15	NC	0	NC	0	NC	0
	D29	5.16	1	NC	0	NC	0	D29	NC	0	NC	0	NC	0
IL-4	D-5	NC	0	NC	0	NC	0	D-6	NC	0	NC	0	NC	0
	D8	NC	0	NC	0	NC	0	D8	NC	0	NC	0	NC	0
	D15	NC	0	NC	0	NC	0	D15	NC	0	NC	0	NC	0
	D29	NC	0	NC	0	NC	0	D29	NC	0	NC	0	NC	0
IL-5	D-5	NC	0	1.06	2	NC	0	D-6	NC	0	0.67	2	NC	0
	D8	0.41	1	0.51	2	0.33	1	D8	0.61	1	0.39	2	0.52	2
	D15	0.41	1	0.53	2	0.44	2	D15	NC	0	0.46	2	NC	0
	D29	0.90	1	NC	0	NC	0	D29	NC	0	NC	0	NC	0
IL-6	D-5	2.56	5	5.40	5	3.38	5	D-6	7.58	5	7.73	5	4.38	5
	D8	1.90	5	3.22	5	1.94	5	D8	2.72	5	3.42	5	3.46	5
	D15	2.33	5	4.90	5	3.01	5	D15	5.26	5	5.59	5	7.79	5
	D29	2.28	2	2.23	2	1.72	2	D29	93.96	2	13.50	2	2.60	2
TNF-α	D-5	0.83	1	NC	0	0.43	1	D-6	0.47	2	0.52	2	0.69	2
	D8	0.62	3	0.56	2	NC	0	D8	NC	0	NC	0	NC	0
	D15	1.05	3	0.54	2	1.01	2	D15	0.72	2	NC	0	NC	0
	D29	1.64	1	0.70	2	0.52	1	D29	NC	0	NC	0	NC	0
IFN-γ	D-5	NC	0	NC	0	NC	0	D-6	NC	0	NC	0	NC	0
	D8	NC	0	NC	0	NC	0	D8	NC	0	NC	0	NC	0
	D15	NC	0	NC	0	NC	0	D15	NC	0	NC	0	NC	0
	D29	NC	0	NC	0	NC	0	D29	NC	0	NC	0	NC	0

[1] Cytokines: serum cytokine levels were measured using CBA kits by Flow Cytometry. The detection limits of IL-2, IL-4, IL-5, IL-6, TNF-α and IFN-γ are 3.6, 0.9, 0.3, 0.1, 0.4 and 3.3 pg/mL, respectively.

[2] Animal numbers for calculation: number of animals with detectable cytokine levels were used for mean values calculation.

[3] NC: not calculable, values were below the detection limit.

Supplementary Table 4

a

Histopathological findings in rats (Terminal Sacrifice, D16)

Tissue	Histopathological findings; Region	Number of animals (Grade[1]) Fresh air (n=20)	HH-120 15 mg/kg (n=20)	HH-120 related[2] Y/N
Adrenal glands	Mineralization; medulla, corticomedullary junction	1 (minimal)	0	N
Harderian glands	Inflammatory cells infiltration; interstitium	1 (slight)	0	N
	Acinar cells degeneration	1 (minimal)	0	N
Kidneys	Cast; cortex	1 (minimal)	0	N
	Mineralization; medulla	1 (minimal)	0	N
	Tubular basophilia; cortex	7 (minimal)	4 (minimal)	N
	Tubular dilatation; cortex	0	1 (minimal)	N
	Mononuclear cells infiltration; cortex	1 (minimal)	1 (minimal)	N
	Mononuclear cells infiltration; medulla	1 (minimal)	0	N
Lungs with bronchi	Alverolar macrophage aggregation	2 (minimal)	0	N
	Osseous metaplasia	2 (minimal)	0	N
	Mononuclear cells infiltration; perivascular	0	1 (slight)	N
Prostate gland[3]	Mononuclear cells infiltration; dorsallobe, interstitum	2 (minimal)	1 (minimal)	N
	Mononuclear cells infiltration; ventrallobe, interstitum	1 (minimal)	0	N

b

Histopathological findings in rats (Recovery Sacrifice, D29)

Tissue	Histopathological findings; Region	Number of animals (Grade[1]) Fresh air (n=10)	HH-120 15 mg/kg (n=10)	HH-120 related[2] Y/N
Heart	Mononuclear cells infiltration; endocardium	0	1 (minimal)	N
	Mononuclear cells infiltration; myocardium	1 (minimal)	0	N
Kidneys	Infarction; cortex	1 (minimal)	0	N
	Mineralization; medulla	0	1 (minimal)	N
	Tubular basophilia; cortex	1 (minimal)	3 (minimal)	N
	Tubular dilatation; cortex, medulla	0	1 (minimal)	N
	Mononuclear cells infiltration; cortex	0	2 (minimal)	N
Liver	Tension lipidosis; hepatocyte	1 (minimal)	0	N
Lungs with bronchi	Osseous metaplasia	0	1 (minimal)	N
	Inflammatory cells infiltration; alveolus	1 (minimal)	0	N
	Inflammatory cells infiltration; alveolus, perivascular	0	1 (minimal)	N
Spleen	Increased extramedullary hemopoiesis; red pulp	5 (minimal)	2 (minimal)	N

[1] Grade: the severity levels of histopathologic grades were evaluated by a certified pathologist, peer reviewed by a JSTP board certified pathologist and assigned as minimal, slight, moderate, marked, severe based on an increasing extent and/or complexity of change.
[2] HH-120 related Y/N: "Y" indicates the finding was HH-120 related, "N" indicates the finding was not HH-120 related. The histological findings were evaluated along with other study parameters, including clinical observations, body weight, organ weight and laboratory tests, and their relevance to HH-120 inhalation was determined with dose dependent incidences and/or common occurrences as spontaneous/background observations.
[3] Prostate gland: prostate gland was evaluated only in male rats.

Supplementary Table 5

a

Histopathological findings in monkeys (Terminal Sacrifice, D15)

Tissue	Histopathological findings; Region	Number of animals (Grade[1])			HH-120 related[2]
		Fresh air (n=6)	HH-120 2.5 mg/kg (n=6)	HH-120 8 mg/kg (n=6)	Y/N
Sternal bone marrow	Granuloma	0	1 (minimal)	0	N
Nasal cavity	Inflammatory cells infiltration; mucosa, submucosa	1 (minimal)	1 (minimal)	2 (1 minimal + 1 slight)	N
	Inflammation exudation; lumen	1 (minimal)	1 (minimal)	0	N
	Vacuolation; respiratory epithelium	3 (minimal)	3 (minimal)	2 (1 minimal + 1 slight)	N
	Vacuolation; respiratory epithelium, olfactory epithelium	0	0	1 (slight)	N
	Degeneration; olfactory epithelium, respiratory epithelium;	0	0	1 (minimal)	N
	Degeneration; subepithelial glands, bowman's glands;	0	0	1 (slight)	N
	Papillary hyperplasia; squamous epithelium	0	0	1 (minimal)	N
Kidneys	Mononuclear cells infiltration; cortex	2 (1 minimal + 1 slight)	2 (minimal)	1 (minimal)	N
Liver	Inflammatory cells infiltration; sinusoid	0	0	2 (minimal)	N
	Mononuclear cells infiltration	0	3 (minimal)	0	N
Lungs with bronchi	Alveolar macrophage aggregation; alveolus	1 (minimal)	0	1 (minimal)	N
	Mononuclear cells infiltration; interstitum	1 (minimal)	0	0	N
	Mononuclear cells infiltration; perivascular, interstitum;	0	1 (slight)	5 (1 minimal + 3 slight + 1 moderate)	Y
	Pigmentation; alveolus	0	0	1 (minimal)	N
	Hemorrhage; alveolus	1 (minimal)	2 (1 minimal + 1 moderate)	1 (minimal)	N
	Bronchiolar epithelial metaplasia; alveolus	0	1 (minimal)	0	N
Ovaries[3]	Mineralization; cortex	1 (slight)	1 (minimal)	1 (minimal)	N
Mandibular salivary gland	Mononuclear cells infiltration; interstitum	0	0	1 (minimal)	N
Skin	Ulceration; subcutaneous	0	0	1 (slight)	N
	Scab; epidermis	0	0	1 (slight)	N
	Edema; subcutaneous	0	0	1 (slight)	N
Stomach	Necrosis; glandular	1 (minimal)	0	1 (minimal)	N
Parotid salivary gland	Mononuclear cells infiltration; interstitum	0	1 (minimal)	0	N

b

Histopathological findings in monkeys (Recovery Sacrifice, D29)

Tissue	Histopathological findings; Region	Number of animals (Grade[1])			HH-120 related[2]
		Fresh air (n=4)	HH-120 2.5 mg/kg (n=4)	HH-120 8 mg/kg (n=4)	Y/N
Adrenal glands	Mineralization; cortex	0	2 (1 minimal + 1 moderate)	1 (moderate)	N
Nasal cavity	Inflammatory cells infiltration; mucosa, submucosa	2 (1 minimal + 1 slight)	3 (2 minimal + 1 slight)	3 (2 minimal + 1 slight)	N
	Inflammation exudation; lumen	1 (slight)	3 (minimal)	3 (2 minimal + 1 slight)	N
	Vacuolation; respiratory epithelium	2 (minimal)	4 (2 minimal + 2 slight)	3 (minimal)	N
	Vacuolation; respiratory epithelium, olfactory epithelium	0	0	1 (slight)	N
	Degeneration; subepithelial glands, bowman's glands	0	0	1 (slight)	N
	Degeneration; bowman's glands	0	0	1 (minimal)	N
Kidneys	Mononuclear cells infiltration; cortex	0	0	2 (1 minimal + 1 slight)	N
Liver	Degeneration; hepatocyte	0	0	1 (minimal)	N
	Mononuclear cells infiltration	0	1 (minimal)	3 (2 minimal + 1 slight)	N
Lungs with bronchi	Fibrosis; alveolus, interstitum	1 (minimal)	0	0	N
	Inflammatory cells infiltration; alveolus	0	0	1 (minimal)	N
	Hemorrhage; alveolus	0	0	1 (minimal)	N
	Neutrophils infiltration; alveolus, interstitum	1 (marked)	0	0	N
	Multinucleated giant cells granuloma; interstitum	0	1 (slight)	0	N
	Macrophage aggregation; alveolus	0	1 (minimal)	0	N
Bronchial lymph node	Macrophage aggregation; medulla	0	0	1 (slight)	N
Ovaries[3]	Mineralization; cortex	0	1 (slight)	0	N
Sublingual salivary gland	Mononuclear cells infiltration; interstitum	0	0	1 (minimal)	N

[1] Grade: The severity levels of histopathologic grades were evaluated by a certified pathologist, peer reviewed by a JSTP board certified pathologist and assigned as minimal, slight, moderate, marked, severe based on an increasing extent and/or complexity of change.

[2] HH-120 related Y/N: "Y" indicates the finding was HH-120 related, "N" indicates the finding was not HH-120 related. The histological findings were evaluated along with other study parameters, including clinical observations, body weight, organ weight and laboratory tests, and their relevance to HH-120 inhalation was determined with dose dependent incidences and/or common occurrences as spontaneous/background observations.

[3] Ovaries: ovaries were evaluated only in female monkeys.

Supplementary Table 6

Histopathology grading Criteria

Severity Levels	Criteria
Minimal	The change is barely discernible and/or very few/very small foci or areas affected.
Slight	the change is more noticeable but only evident as few/small foci or areas affected.
Moderate	The change is obviously present, and of appreciable size and/or number.
Marked	The change is abundant in many areas of the section and/or is of prominent size.
Severe	The change affects a large proportion of the tissue and/or is of a large size.

Supplementary Figure 1

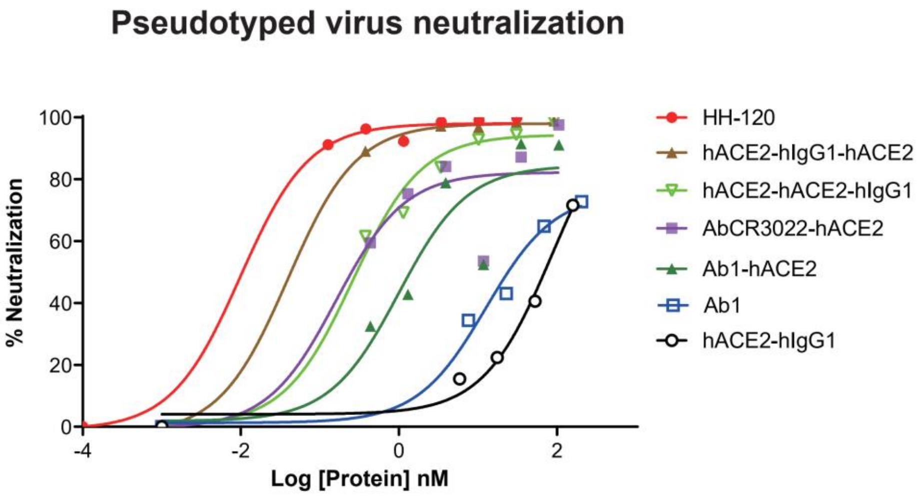

Supplementary Figure 1. Pseudotyped virus neutralization analysis of HH-120 and other ACE2 fusion proteins or antibodies. Neutralization assays were conducted using SARS-CoV-2 D614 pseudotyped virus (lentivirus system). Pseudotyped viruses were incubated with serially diluted proteins, then inoculated with 293T-hACE2 cells. hACE2-hACE2-hIgG1 was constructed by tandemly fusing two hACE2 proteins to the N terminus of hIgG1. hACE2-hIgG1-hACE2 was constructed by fusing hACE2 to the N terminus and the C terminus of hIgG1. AbCR3022-hACE2, Ab1-hACE2 were constructed by fusing hACE2 to the C terminus of CR3022 and Ab1. AbCR3022 is a monoclonal antibody cross-react with RBDs of SARS-CoV-2 and SARS-CoV[66]. Ab1 is a SARS-CoV-2 neutralization antibody.

Supplementary Figure 2

Alpha

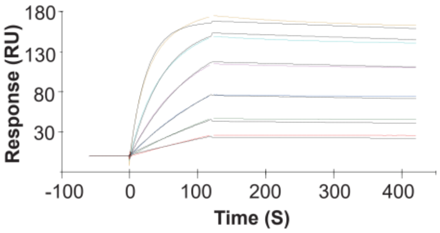

KD (M)	Ka(1/Ms)	Kd(1/s)
9.5E-10	1.98E+05	1.88E-04

Beta

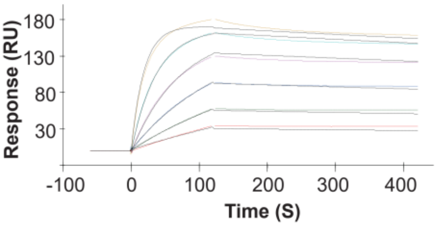

KD (M)	Ka(1/Ms)	Kd(1/s)
1.13E-09	2.69E+05	3.05E-04

Delta

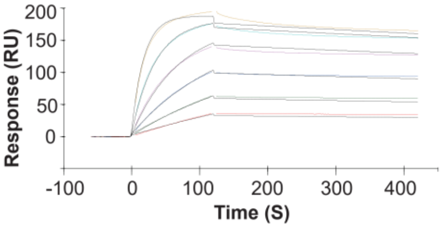

KD (M)	Ka(1/Ms)	Kd(1/s)
1.17E-09	2.79E+05	3.26E-04

Omicron

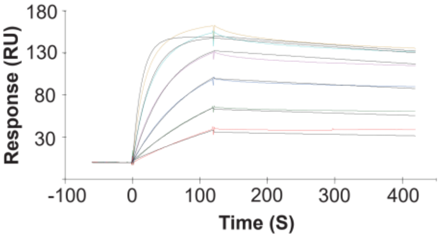

KD (M)	Ka(1/Ms)	Kd(1/s)
1.14E-09	3.72E+05	4.23E-04

Supplementary Figure 2. Affinity and kinetic characterization of the binding of HH-120 to SARS-CoV-2 S trimer proteins. The S trimer proteins of Alpha, Beta, Delta, and Omicron variants were analyzed for binding to HH-120 using a Biacore T200 instrument.

Supplementary Figure 3

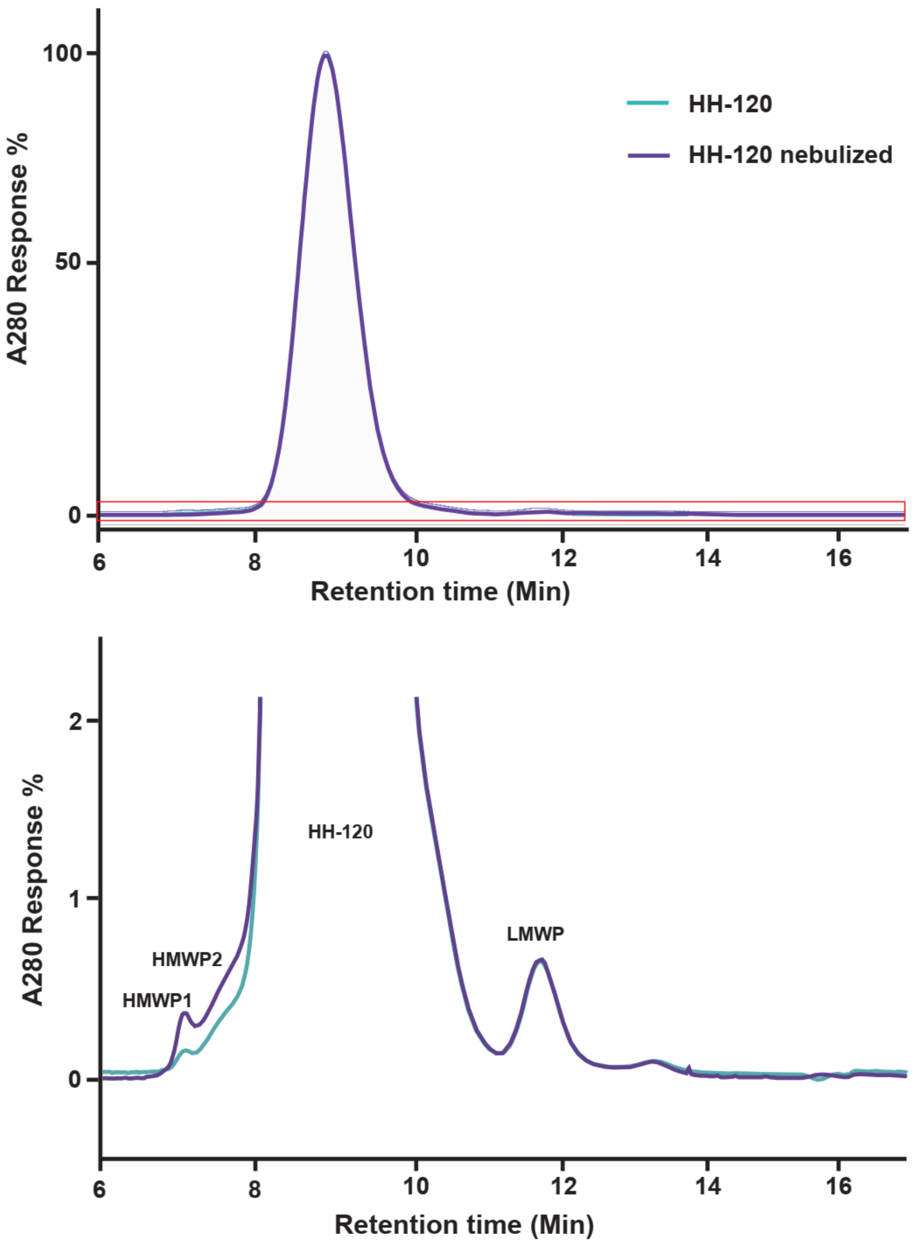

Percentage (%) of each component before and after nebulization

	HMWP1	HMWP2	HH-120	LMWP
HH-120	0.06	0.25	99.13	0.51
HH-120 nebulized	0.18	0.41	98.78	0.56

Supplementary Figure 3. SEC-HPLC analysis of HH-120 and nebulized HH-120. 100 μg of HH-120 or nebulized HH-120 samples were assessed on a TSKgel G4000SWxl analytical column using an Agilent 1260 HPLC system. The lower panel presents the magnification of the red box zone in the upper HPLC chromatogram. HMWP1, HMWP2, HH-120, and LMWP components are indicated. The table shows the percentage of each component.

Supplementary Figure 4

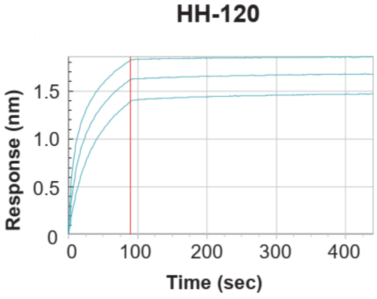

KD (M)	Ka(1/Ms)	Kd(1/s)
<1.0E-12	3.93E+05	<1.0E-07

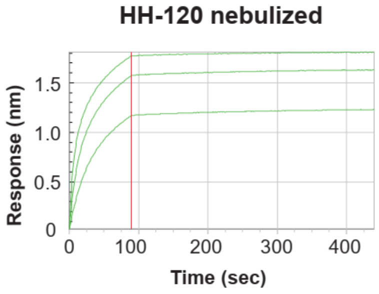

KD (M)	Ka(1/Ms)	Kd(1/s)
<1.0E-12	3.89E+05	<1.0E-07

Supplementary Figure 4. Avidity and kinetic characterization of the binding of HH-120 or nebulized HH-120 to SARS-CoV-2 RBD. The binding avidity and kinetics were measured using a Fortebio RED384 instrument.

Supplementary Figure 5

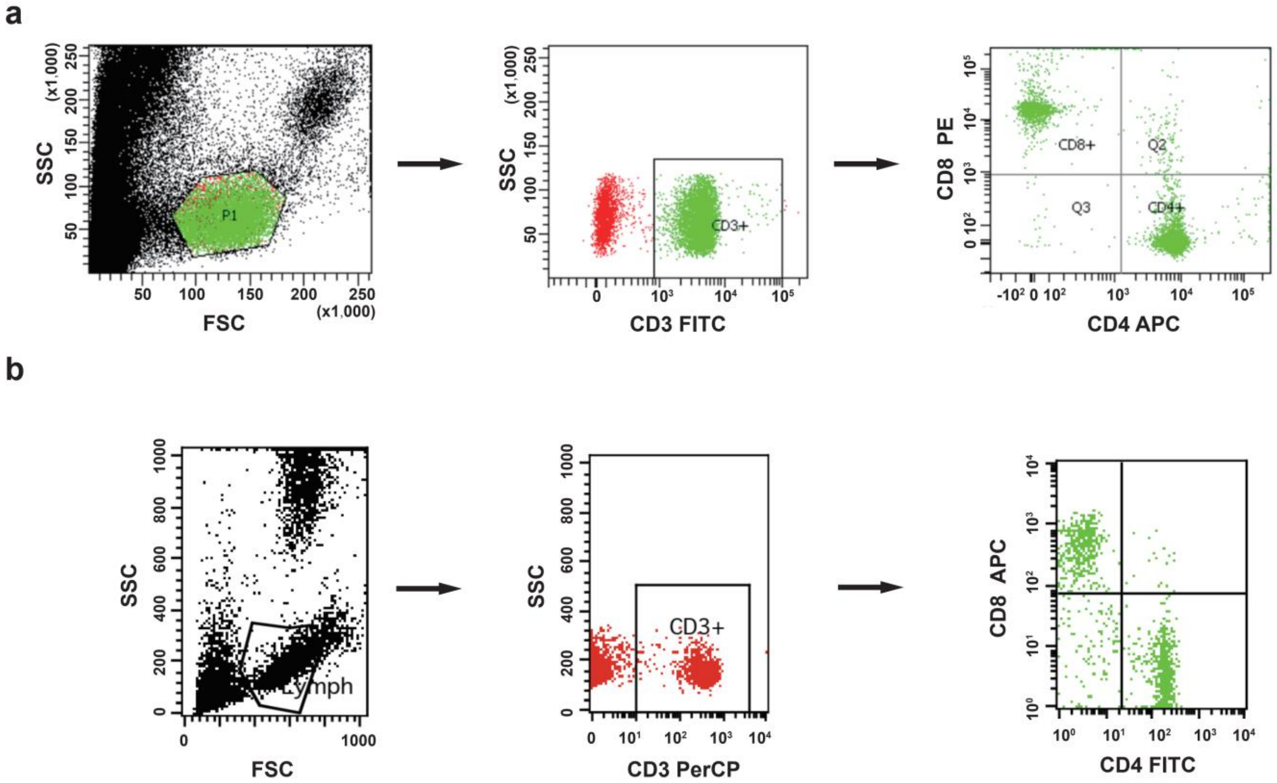

Supplementary Figure 5. Gating strategies for defining T lymphocyte subsets in the toxicology studies. a, Gating strategies for the FACS data panel shown in Figure 6d to define $CD3^+$, $CD3^+CD4^+$, $CD3^+CD8^+$ T lymphocyte subsets in SD rats in the two-week repeated dose toxicology study. **b**, Gating strategies for the FACS data panel shown in Figure 6h to define $CD3^+$, $CD3^+CD4^+$, $CD3^+CD8^+$ T lymphocyte subsets in monkeys in the two-week repeated dose toxicology study.
